# A probabilistic framework for cellular lineage reconstruction using integrated single-cell 5-hydroxymethylcytosine and genomic DNA sequencing

**DOI:** 10.1016/j.crmeth.2021.100060

**Published:** 2021-07-30

**Authors:** Chatarin Wangsanuwat, Alex Chialastri, Javier F. Aldeguer, Nicolas C. Rivron, Siddharth S. Dey

**Affiliations:** 1 Department of Chemical Engineering, University of California Santa Barbara, Santa Barbara, CA 93106, USA; 2 Center for Bioengineering, University of California Santa Barbara, Santa Barbara, CA 93106, USA; 3 Hubrecht Institute – KNAW and University Medical Center Utrecht, Utrecht, the Netherlands; 4 Institute of Molecular Biotechnology of the Austrian Academy of Sciences (IMBA), Vienna BioCenter (VBC), Vienna, Austria; 5 Neuroscience Research Institute, University of California Santa Barbara, Santa Barbara, CA 93106, USA

**Keywords:** lineage reconstruction, individual-cell-division resolution, 5-hydroxymethylcytosine, integrated single-cell genomic DNA and 5-hydroxymethylcytosine sequencing, preimplantation mouse embryogenesis, immortal strand hypothesis

## Abstract

Lineage reconstruction is central to understanding tissue development and maintenance. To overcome the limitations of current techniques that typically reconstruct clonal trees using genetically encoded reporters, we report scPECLR, a probabilistic algorithm to endogenously infer lineage trees at a single-cell-division resolution by using 5-hydroxymethylcytosine (5hmC). When applied to 8-cell pre-implantation mouse embryos, scPECLR predicts the full lineage tree with greater than 95% accuracy. In addition, we developed scH&G-seq to sequence both 5hmC and genomic DNA from the same cell. Given that genomic DNA sequencing yields information on both copy number variations and single-nucleotide polymorphisms, when combined with scPECLR it enables more accurate lineage reconstruction of larger trees. Finally, we show that scPECLR can also be used to map chromosome strand segregation patterns during cell division, thereby providing a strategy to test the “immortal strand” hypothesis. Thus, scPECLR provides a generalized method to endogenously reconstruct lineage trees at an individual-cell-division resolution.

## Highlights

• scPECLR uses 5hmC to endogenously reconstruct lineage trees
• scPECLR reconstructs lineage trees at an individual-cell-division resolution
• Combined single-cell 5hmC and gDNA sequencing allows reconstruction of larger trees
• The method can be used to map DNA strand segregation patterns during cell division

## Motivation

Reconstructing lineage trees is fundamental for gaining insights into basic biological and disease processes. Although powerful tools to infer cellular relationships have been developed, these methods typically have a clonal resolution that prevents the reconstruction of lineage trees at an individual-cell-division resolution. Moreover, these methods require a transgene, which poses a significant barrier to the study of human tissues. In this work, we develop a complementary approach that does not require exogenous labeling and can reconstruct each cell division within a lineage tree.

## Graphical abstract

**Figure:**
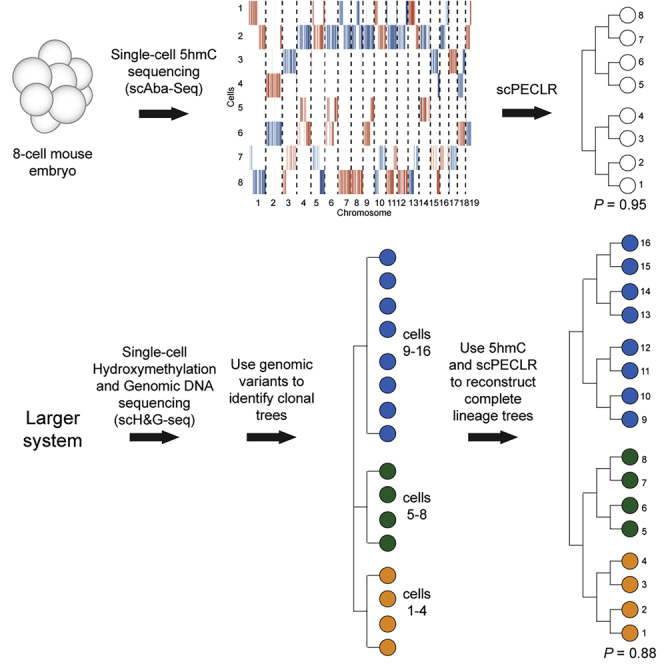

## Teaser

Wangsanuwat et al. develop a probabilistic algorithm to reconstruct cellular lineages at an individual-cell-division resolution by using strand-specific 5hmC measurements in single cells, and combine it with the development of a single-cell multi-omics technology to quantify 5hmC and genomic DNA from the same cell to reconstruct larger lineage trees.

## Introduction

Understanding lineage relationships between cells in a tissue is a central question in biology. Reconstructing lineage trees is not only fundamental to understanding tissue development, homeostasis, and repair but is also important for gaining insights into the dynamics of tumor evolution and other diseases. Genetically encoded fluorescent reporters have been a powerful approach to reconstruct the lineage of many tissues (Kretzschmar and Watt, 2012). However, these methods require the generation of complex animal models for each stem or progenitor cell type of interest, and are limited to a clonal resolution (Kretzschmar and Watt, 2012). Similarly, other techniques, such as the use of viruses (Naik et al., 2013), transposons (Sun et al., 2014; Wagner et al., 2018), Cre-loxP-based recombination (Pei et al., 2017), and CRISPR/Cas9 (Alemany et al., 2018; Kalhor et al., 2017; McKenna et al., 2016; Perli et al., 2016; Raj et al., 2018; Spanjaard et al., 2018) have also been used to genetically label cells to primarily reconstruct clonal lineages. This clonal resolution limits our ability to understand tissue dynamics at a single-cell-division resolution. Although a recent report that combined CRISPR/Cas9-mediated mutagenesis with single-molecule RNA fluorescence *in situ* hybridization (FISH) enabled reconstruction of lineages at a single-cell-division resolution (MEMOIR) (Frieda et al., 2017), their ability to infer lineages dropped substantially by the third cell division.

Furthermore, as these methods involve exogenous labeling, they cannot be used to directly map cellular lineages in human tissues, thereby posing a barrier to understanding human development and diseases. Although endogenous somatic mutations have been used to reconstruct lineages, their low frequency of occurrence over the whole genome make them challenging to detect and therefore limit their application as a lineage reconstruction tool (Behjati et al., 2014; Ju et al., 2017; Lodato et al., 2015). Similarly, recent methods have used mutations within the mitochondrial genome or microsatellites to reconstruct lineages, but these approaches are also limited to a clonal resolution (Biezuner et al., 2016; Evrony et al., 2015; Ludwig et al., 2019; Xu et al., 2019). Previously, we developed a method to detect the endogenous epigenetic mark 5-hydroxymethylcytosine (5hmC) in single cells (scAba-seq) and showed that the lack of maintenance of this mark during replication resulted in older DNA strands containing higher levels of 5hmC (Mooijman et al., 2016). The ability to track individual DNA strands through cell division allowed us to deterministically reconstruct lineages that were limited to two cell divisions (Mooijman et al., 2016). Therefore, to reconstruct larger trees and overcome limitations of other methods, we report single-cell Probabilistic Endogenous Cellular Lineage Reconstruction (scPECLR), a generalized probabilistic framework to endogenously reconstruct cellular lineages at an individual-cell-division resolution by using single-cell 5hmC sequencing. This approach can be used to successfully reconstruct up to four cell divisions. To reconstruct larger trees, we developed an integrated single-cell method, scH&G-seq, to simultaneously sequence 5hmC and genomic/mitochondrial DNA from the same cell. By combining information from genomic variants that can be used to identify clonal subtrees within the complete tree, together with strand-specific 5hmC that enables tracking the lineage of individual cells, scH&G-seq can be generalized to endogenously reconstruct the lineage of larger trees at a single-cell-division resolution.

## Results

### Genome-wide strand-specific 5hmC enables initial lineage bifurcation of individual cells into two subtrees

**Figure 1:**
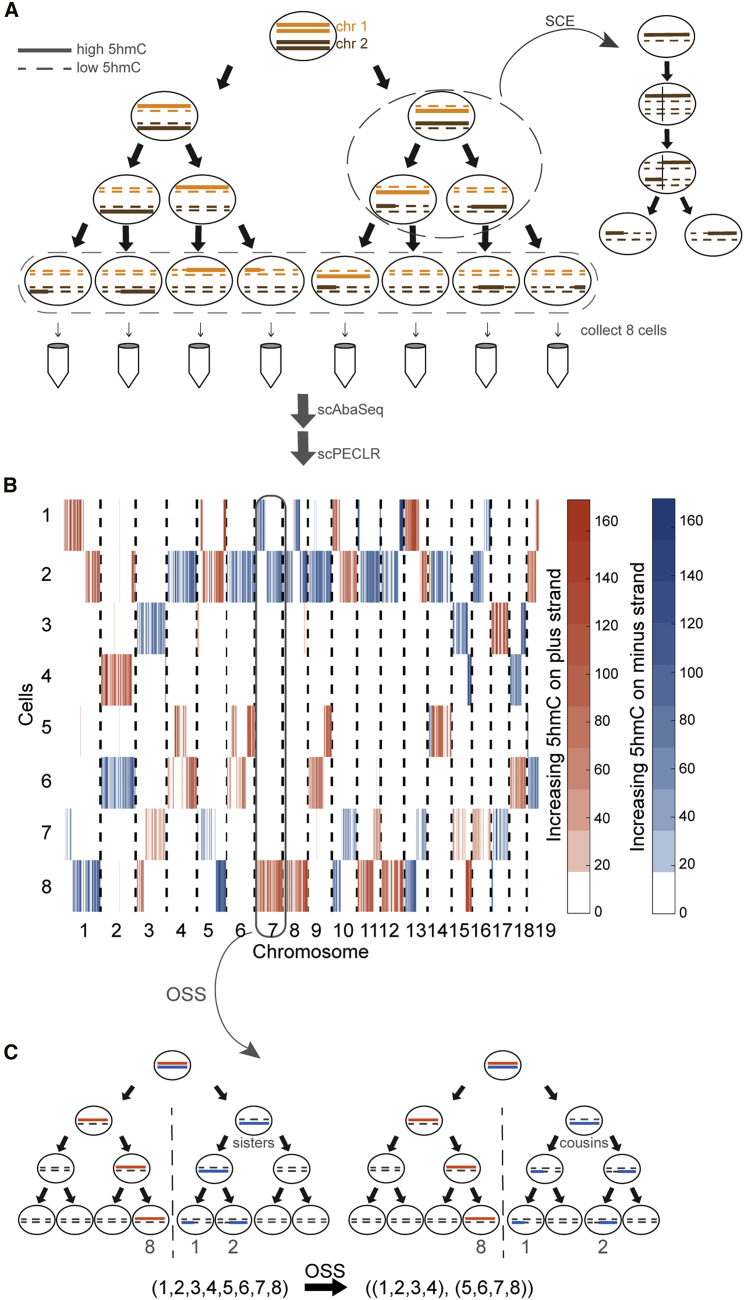
Strand-specific single-cell 5hmC enables initial lineage bifurcation of individual cells into two subtrees (A) Schematic shows a zygote with chromosomes containing high 5hmC levels (solid lines) undergoing three cell divisions. The newly synthesized strands contain very low levels of 5hmC (dotted lines). SCE events occur randomly during each cell cycle. Single cells are sequenced by using scAba-seq to quantify strand-specific 5hmC. (B) Data showing mosaic pattern of strand-specific 5hmC in single cells obtained from an 8-cell mouse embryo. 5hmC counts within 2-Mb bins on the plus and minus strands are shown in orange and blue, respectively. (C) OSS analysis on chromosome 7 places cell 8 in one 4-cell subtree and cells 1 and 2 in the other subtree. Performing OSS on all chromosomes places cells in one of these two 4-cell subtrees and reduces the complexity of the lineage reconstruction problem.

As proof of principle, we dissociated 8-cell mouse embryos and performed scAba-seq to quantify strand-specific genome-wide patterns of 5hmC in single cells (Figure 1A). As shown previously, a majority of 5hmC is present on the paternal genome during these stages of pre-implantation development (Inoue and Zhang, 2011; Iqbal et al., 2011; Wossidlo et al., 2011). Single cells from an 8-cell embryo displayed a mosaic genome-wide distribution with no overlap of 5hmC between the plus and minus strands of a chromosome (Figure 1B). Furthermore, for each chromosome the strand-specific 5hmC was localized to a few cells, and other cells contained undetectable levels of the mark (Figure 1B). These observations show that only one allele carries a majority of 5hmC and that we are primarily detecting 5hmC on the original paternal genome, with DNA strands synthesized in subsequent rounds of replication carrying very low levels of the mark. We used this as our basis to reconstruct cellular lineages of 8-cell embryos.

As the first step toward reconstructing lineage trees, we noted that the original plus and minus strands of each paternal chromosome in the 1-cell zygote will be found in distinct cells on opposite sides of the lineage tree after n cell divisions. As a result, all cells can be placed in one of two subtrees, thereby reducing the number of cell divisions to be reconstructed from n to n−1. For example, at the 8-cell stage, the original paternal plus strand of chromosome 7 is detected in cell 8 and the minus strand is detected in cells 1 and 2 (Figure 1B). This suggests that cell 8 is on the opposite side of the tree compared with cells 1 and 2. Performing this first step of scPECLR, referred to as original strand segregation (OSS) analysis, over all the chromosomes enables us to place cells 1–4 and 5–8 on opposite sides of the lineage tree, reducing the complexity of the problem from reconstructing 3 cell divisions with 315 tree topologies to 2 cell divisions with 9 tree topologies (Figure 1C).

### Probabilistic lineage reconstruction using scPECLR accurately predicts 8-cell embryo trees

To reconstruct the complete lineage tree, we next used the mosaic pattern of 5hmC arising from abrupt transitions in hydroxymethylation levels among cells along the length of a chromosome. These sharp transitions in 5hmC that are shared between two cells are the result of homologous recombination during sister chromatid exchange (SCE) events in the G_2_ phase of a previous cell cycle (Mooijman et al., 2016). Detection of 5hmC transitions that are common to two cells therefore indicate a shared evolutionary history between these cells (Figure 1A, inset). However, although an SCE event at the 4-cell stage would imply that the cells are sisters (Figure 1C, left), one occurring at the 2-cell stage would indicate that the same pattern of 5hmC transition can also be observed between cousins (Figure 1C, right). Thus, the observation of a single shared SCE event between two cells cannot be used to immediately discriminate between sister and cousin cell configurations.

**Figure 2:**
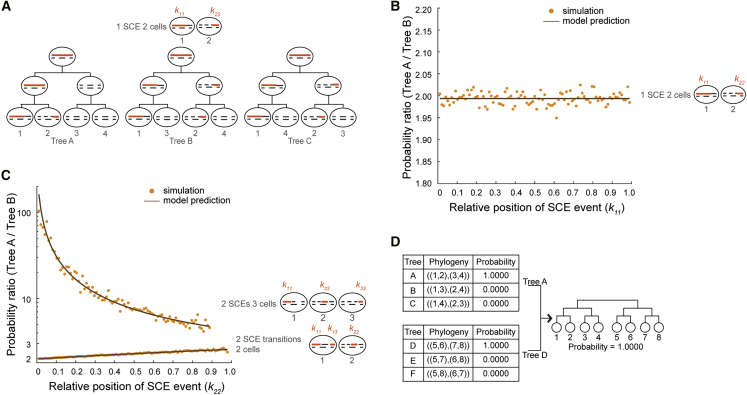
Endogenous 5hmC-based lineage reconstruction using scPECLR (A) Two cells sharing an original DNA strand (solid orange line) can either be sisters (Tree A) or cousins (trees B and C) depending on whether the SCE event occurred at the 4- to 8-cell or 2- to 4-cell stage, respectively. Newly synthesized DNA strands are shown as dashed black lines. (B) For an SCE transition between two cells, the probability of the pair of cells being sisters versus cousins is plotted against the relative position of the SCE event on the chromosome ($k_{11}$). The model prediction (black) and simulation results (yellow) are shown for chromosome 1 (*N* = 97 for 2-Mb bins) with b=0.3. (C) The probability ratio between Trees A and B are shown for *N* = 97 and b=0.3 for two cases: two SCE transitions shared between two cells and two SCE events shared between three cells. (D) For the 8-cell mouse embryo in Figure 1B, the probability of observing the different topologies, rounded to four decimal places, for the two 4-cell subtrees are shown.

To systematically determine the likelihood of observing different tree topologies, we developed a probabilistic framework where the occurrence of SCE events is modeled as a Poisson process. The total number of SCE events is used to estimate the parameter b of the Poisson process, the rate of SCE events per chromosome per cell division, using maximum-likelihood estimation (STAR Methods). After OSS, 8-cell trees can be grouped into two 4-cell subtrees, each with three possible tree arrangements (Figure 2A). Next, we used the probabilistic model to calculate the likelihood of observing an SCE pattern for a chromosome given a tree topology. We observed a large variety of SCE patterns, ranging from commonly observed patterns, such as one or two SCE transitions shared between two cells, to more complex distributions of 5hmC between cells (Figure S1). For the most common pattern of one SCE transition between two cells, scPECLR predicts that the tree with the two cells as sisters (tree A) is twice as likely as one where the two cells are cousins (tree B or C), in good agreement with simulated data (Figure 2B and STAR Methods). Similarly, when two SCE transitions are shared between two cells, the probability that the two cells are sisters is 2–3 times higher than the probability that they are cousins, with the likelihood ratio between sister and cousin tree configurations depending on the relative position of the SCE transition on the chromosome (Figures 2C and S2; STAR Methods). More complex 5hmC distribution patterns, such as when two SCE events are shared between three cells, substantially favors the configuration of tree A (Figure 2C and STAR Methods). After the SCE pattern of each chromosome is analyzed, we can estimate the total likelihood of observing different tree topologies, assuming that the SCE events on each chromosome are independent (STAR Methods). Finally, the likelihood of an 8-cell tree is the product of the likelihoods of the two corresponding 4-cell subtrees (Figure 2D and Method S1).

**Figure 3:**
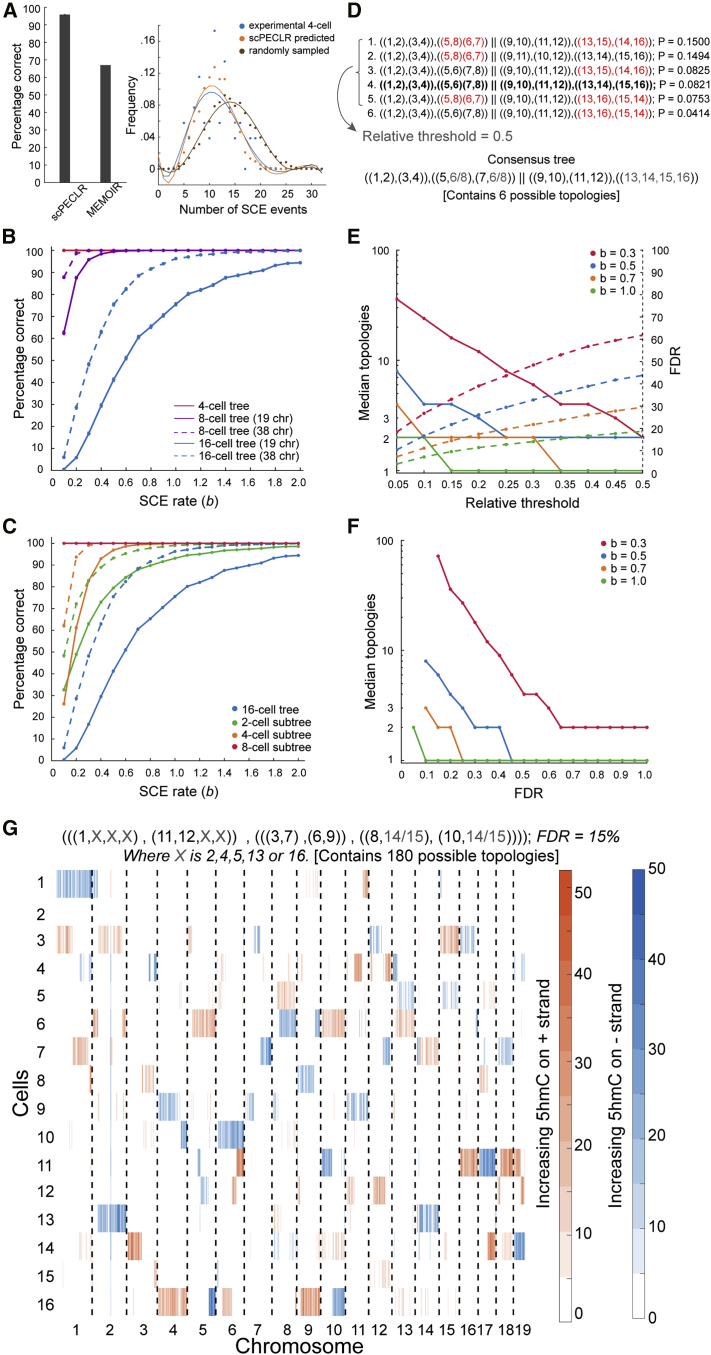
scPECLR can reconstruct 8- and 16-cell lineage trees (A) (Left) scPECLR accurately predicts the lineage of 96% of simulated 8-cell trees (b=0.3). Error bars indicate the bootstrapped standard error. In comparison, MEMOIR accurately predicts 67% of the top 40% most reliably reconstructed 8-cell trees (Frieda et al., 2017). (Right) The distribution of SCE events in 4-cell embryos (blue) is not statistically different from that of 4-cell trees inferred with scPECLR starting from 8-cell trees (orange, p > 0.8), but is different from 4-cell trees inferred from a random topology at the 8-cell stage (brown, $\text{p}<10^{-4}$). (B) Percentage of simulated 8- and 16-cell trees that are correctly predicted by scPECLR for different SCE rates (b). The prediction accuracy is computed by simulating 5,000 trees. Error bars indicate the bootstrapped standard error. (C) Percentage of 2-, 4-, and 8-cell subtrees that are accurately predicted within simulated 16-cell trees as a function of the SCE rate (b). The prediction accuracy is computed by simulating 5,000 16-cell trees. Error bars indicate the bootstrapped standard error. (D) Construction of consensus trees. In this example, the top six tree topologies (with the highest probabilities) obtained after applying scPECLR on a 16-cell tree are shown. The relative threshold (RT) parameter is used to determine the number of topologies considered in the consensus tree analysis. With an RT of 0.5, the top 5 topologies are selected to generate a consensus tree that is consistent with all these trees. The uncertainty within the consensus tree is quantified by the number of tree topologies it contains. Red fonts indicate parts of the lineage tree that are incorrectly predicted. The tree highlighted in bold is the true tree. (E) Simulations show that as the RT increases, the median number of topologies in the consensus tree decreases (solid lines, left axis) whereas the false discovery rate (FDR) increases (dotted lines, right axis). In these simulations, two other parameters t8 and t4 are set to 0.75 and 1.0, respectively. For details, see STAR Methods. (F) Graph showing how the specificity of the consensus tree is related to error tolerance. As the FDR decreases, the median number of topologies contained within the consensus tree increases. Note that the lowest FDR possible for b = 0.3, 0.5, 0.7, and 1.0 are 15%, 10%, 10%, and 5%, respectively. (G) Single-cell 5hmC sequencing data for a 16-cell mouse embryo (4-Mb bins). The consensus tree associated with this embryo is estimated to have a 15% FDR rate. RT, t8, and t4 are set at 0.05, 0.85, and 0.8, respectively. The consensus tree is constrained to only 180 possible topologies, a significant reduction from the more than 600 million trees originally.

To test the accuracy of scPECLR, we simulated 5hmC patterns of 8-cell embryos with an SCE rate similar to the experimentally observed value (b=0.3) and within the range found in other cell types (Falconer et al., 2012; Hongslo et al., 1991; Tateishi et al., 2003; Wu et al., 2017; Zack et al., 1977). scPECLR predicted the lineage tree correctly in 96% of all simulations (Figure 3A, left). In contrast, MEMOIR predicted the lineage tree accurately in only ∼67% of the top 40% most reliably reconstructed trees, although this was based on ground truth obtained from imaging data (Figure 3A, left). This improved accuracy of scPECLR strongly suggests that endogenous strand-specific 5hmC patterns present an accurate tool to reconstruct lineage trees at an individual-cell-division resolution. Furthermore, to directly validate our method against experimental data, we combined the lineage trees predicted by scPECLR from simulated 8-cell embryos to estimate the number of SCE events at the 4-cell stage. We hypothesized that if scPECLR predicted the correct tree then it would produce a distribution of SCE events similar to that of the experimental data at the 4-cell stage. We found that the scPECLR-predicted distribution of SCE events per cell at the 4-cell stage was statistically indistinguishable from the experimentally obtained distribution in 4-cell embryos (p>0.8, two-sample Kolmogorov-Smirnov [KS] test) (Figure 3A, right). In contrast, when one of the 314 incorrect tree topologies at the 8-cell stage were sampled randomly, it resulted in a distribution of SCE events per cell that was significantly different from the experimental data ($\text{p}<10^{-4}$, two-sample KS test) (Figure 3A, right). These results show that scPECLR can reconstruct three cell divisions with high accuracy. Finally, we applied scPECLR on the 8-cell mouse embryo shown in Figure 1B and other embryos to predict lineage trees with high confidence (Figure 2D and Data S1).

As SCE transitions play a central role in reconstructing lineage trees with scPECLR, we next explored how the endogenous rate of SCE events influences the accuracy of the model. As expected, the accuracy of lineage reconstruction increases monotonically with increasing rates of SCE events, and greater than 98% of the simulated 8-cell trees were correctly predicted for b≥0.4 (Figure 3B and STAR Methods). These simulations were performed by using 19 paternal autosomes based on our observations in pre-implantation mouse embryos; however, most cell types carry 5hmC on both alleles, and therefore we also performed simulations with 38 chromosomes. Again, as expected, the predictive power of the model increases, and more than 98% of the simulated 8-cell trees were accurately predicted for b≥0.2 (Figure 3B). These results demonstrate that the lineage tree can be accurately predicted up to three cell divisions even with low rates of SCE events (Figure 3B).

### scPECLR can be extended to reconstruct the lineage of 16-cell trees

We next extended scPECLR to reconstruct the lineage of 16-cell trees, whereby the number of possible tree topologies increases exponentially to more than 6 × 10^8^. Although the ability to predict the complete lineage tree decreases (17% accuracy for b=0.3), large parts of the tree were reconstructed accurately, with the most common error being the misidentification of one sister pair within a 4-cell subtree (Figures 3B and 3C). For an SCE rate of b=0.3, 83% of all 4-cell subtrees and 63% of all 2-cell subtrees were predicted correctly (Figure 3C). These results suggest that when reconstructing 16-cell trees it is important to identify parts of the tree that can be predicted with high confidence. To accomplish this, we first included all tree topologies with probabilities above a threshold in relation to the tree with the highest probability (Figure 3D). A consensus tree that is consistent with all these tree topologies is then established (Figures 3D and S3; STAR Methods). As the relative threshold is increased (i.e., we include fewer tree topologies to construct the consensus tree), the median consensus tree contains fewer topologies, resulting in a more specific consensus tree. However, this results in an increase in false discovery rate (FDR). For example, with b=0.3 and a relative threshold of 0.1, the median consensus tree contained 24 tree topologies (Figure 3E, solid red line). The consensus trees displayed an FDR of ∼26%, implying that in 26% of the simulations the consensus tree has at least some part of the lineage tree that is not consistent with the true tree (Figure 3E, dotted red line). Thus, the relative threshold allows us to tune the competing goals of specificity and accuracy of the consensus tree. These results show that for a certain rate of SCE events and a desired level of FDR, the median number of topologies contained in the consensus tree can be estimated, yielding insights into how much lineage information can be extracted (Figure 3F and STAR Methods). Finally, as proof of principle, we sequenced a 16-cell mouse embryo and applied scPECLR to show that we can extract partial lineage information from larger trees (Figure 3G and STAR Methods).

### Integrated single-cell genomic DNA and 5hmC sequencing enables reconstruction of larger lineage trees

**Figure 4:**
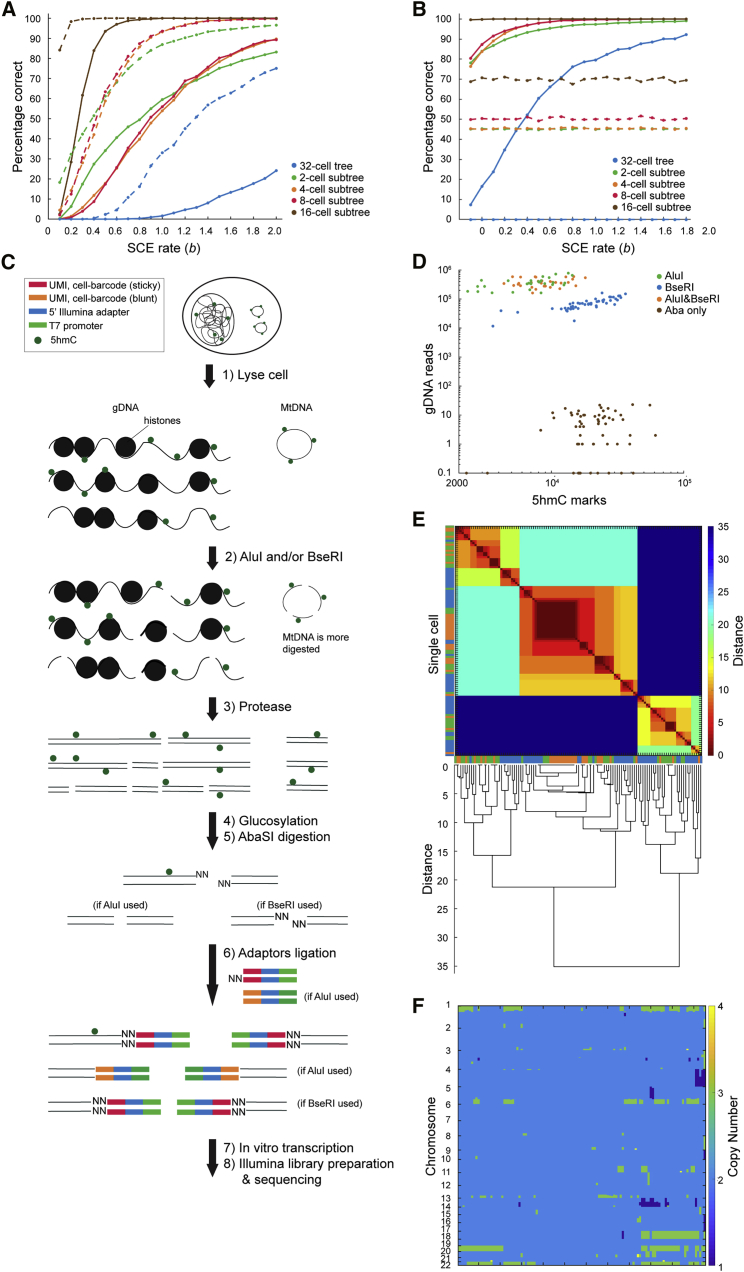
Integrated single-cell 5hmC and genomic DNA sequencing can be used to endogenously reconstruct larger lineage trees (A) Percentage of the full lineage, along with 2-, 4-, 8-, and 16-cell subtrees, that are accurately predicted in simulated 32-cell trees as a function of SCE rates (b). The prediction accuracy is computed by simulating 2,000 trees. Solid and dotted lines indicate cells where 5hmC can be quantified in 19 or 38 chromosomes, respectively. (B) Percentage of the full lineage, along with the subtrees, that are correctly predicted in simulated 32-cell trees as a function of SCE rates (b), by using information from both 5hmC and gDNA. Solid and dotted lines indicate prediction accuracy by using integrated information and gDNA alone, respectively. The prediction accuracy is computed by simulating 2,000 38-chromosome trees, and the rate of occurrence of genomic variants is set to 0.6 per chromosome per cell division. (C) Schematic illustration of scH&G-seq. (D) scH&G-seq enables simultaneous detection of gDNA and 5hmC from the same cell. (E) Heatmap of the Euclidean distance between cells and the corresponding dendrogram. Single cells cluster into two major groups. Cells from AluI, BseRI, and dual enzyme libraries are displayed in green, orange, and blue, respectively. (F) Heatmap of the copy number profile of single cells sorted in the same order as the dendrogram in (E).

For larger 32-cell trees, the number of possible tree topologies increases to more than 10^26^, making it computationally very expensive to calculate the likelihood of all trees. Therefore, we extended scPECLR by developing an algorithm that efficiently searches through the tree topology space to reconstruct these larger trees. After OSS bifurcates the 32 cells into 2 16-cell subtrees, we identify groups of 8 cells that when combined minimize the number of SCE events at the 4-cell stage. This algorithm relies on the strategy that incorrectly grouped cells will increase the number of SCE events at the 4-cell stage, and this subsampling enables rapid search through the tree topology space. Finally, the four groups of eight cells are reconstructed using scPECLR as described above (STAR Methods). As expected, although the ability to predict the complete lineage tree is lower than that for 16-cell trees, this method can rapidly predict subtrees within the 32-cell tree. For example, for b=1 and 19 alleles, 2-, 4-, and 8-cell subtrees are predicted with 50%–60% accuracy, whereas the 16-cell subtrees are predicted at close to 100% accuracy (Figure 4A, solid lines). For the more general case of 38 alleles in mouse genomes, the prediction accuracy increases substantially, and 80%–95% of the 2-, 4-, and 8-cell subtrees were predicted correctly for b=1 (Figure 4A, dotted lines).

To endogenously reconstruct large lineage trees at an individual-cell division resolution, we hypothesized that single-cell strand-specific 5hmC data combined with information on genomic variants, such as genomic copy-number variations (CNV), genomic single-nucleotide polymorphisms (SNPs), or mitochondrial SNPs, could significantly improve the prediction accuracy. Genomic variants have previously been used to reconstruct clonal lineages and, therefore, when integrated with strand-specific 5hmC could help anchor subtrees within the complete lineage tree (Biezuner et al., 2016; Evrony et al., 2015; Ludwig et al., 2019; Xu et al., 2019). To test this hypothesis, we simulated trees with genomic variants together with SCE events and found that the prediction accuracy increases dramatically compared with the use of SCE events alone (Figures 4A, 4B, S4B, and S4C; STAR Methods). For example, for b=1, the complete 32-cell lineage tree was predicted correctly in 76% of all simulations, and the 2- to 16-cell subtrees were predicted with greater than 96% accuracy (Figure 4B). In contrast, when using 5hmC or genomic variants alone, the prediction accuracy was lower (Figures 4A and 4B). Overall, these results demonstrate that 5hmC and genomic variants together present a general strategy to accurately reconstruct large lineage trees at a single-cell division resolution.

To accomplish this goal experimentally, we developed a method to simultaneously quantify 5hmC and the genome from the same cell (scH&G-seq). Single cells are lysed, and the genomic DNA (gDNA) and mitochondrial DNA (mtDNA) are digested by using the restriction enzymes AluI and/or BseRI (Figure 4C). After stripping chromatin from gDNA, 5hmC sites are glucosylated, and these sites are thereafter digested by the restriction enzyme AbaSI (Figure 4C). Double-stranded adapters, containing a cell-specific barcode, a 5′ Illumina adapter, and T7 promoter, together with restriction enzyme-compatible overhangs, are ligated to the fragmented DNA molecules (Figure 4C). These ligated molecules are then amplified by *in vitro* transcription and used to prepare Illumina libraries as described previously (Hashimshony et al., 2016; Mooijman et al., 2016; Rooijers et al., 2019; Sen et al., 2021), enabling simultaneous quantification of gDNA, mtDNA, and 5hmC from the same cell.

As proof of concept, we applied scH&G-seq to single H9 human embryonic stem cells with different combination of restriction enzymes—AluI and AbaSI, BseRI and AbaSI, or AluI, BseRI, and AbaSI—and successfully detected both gDNA/mtDNA and 5hmC from the same cell (Figures 4D and S4D). We detected a similar number of 5hmC sites per cell, when compared with scAba-seq control cells, and integration with additional restriction enzymes enabled genome-wide sequencing of gDNA/mtDNA (Figures 4D and S4D). To show that gDNA variants can infer clonal cellular relationships, we called CNVs in single cells. Hierarchical clustering identified two major clusters with a diploid and non-diploid population, with additional subgroups within the non-diploid population (Figures 4E and 4F). These results demonstrate that scH&G-seq can be used to predict large lineage trees at a single-cell-division resolution. Similarly, the high mutation rate in mtDNA has previously been used to reconstruct clonal lineage trees, and therefore we used scH&G-seq to identify mtSNPs. Although we identified nearly 40 mtSNPs in H9 cells when mapping to the reference human genome, these SNPs were observed at a frequency of close to 100%. Comparison with previously published ATAC-seq data from H9 cells together with SNP calls from another human cell line also identified the same SNPs, suggesting that these nucleotides represented the wild-type sequence (Table S1) (Diroma et al., 2020; Liu et al., 2017). Nevertheless, these results show that in addition to sequencing 5hmC in single cells, scH&G-seq can be used to obtain clonal lineage information that can together be used to reconstruct larger trees.

### scPECLR can be used to infer the rate of SCE events at each cell division and test the “immortal strand” hypothesis

In addition to reconstructing lineage trees, scPECLR can also be used to infer the rate of SCE events at each cell division. For example, in 8-cell embryos, the 5hmC distribution at the 4-cell and 2-cell stages can be reconstituted on the basis of the predicted lineage, enabling us to estimate the rate of SCE events at each cell division (Figure 2D and Data S1). Although the overall SCE rate over three cell divisions for all the 8-cell mouse embryos analyzed in this study was estimated to be 0.35 events per chromosome per cell division on average, the individual SCE rates for the 1- to 2-cell, 2- to 4-cell, and 4- to 8-cell stages were 0.31, 0.24, and 0.51, respectively. Furthermore, we found that the different rates of SCE events at each cell division did not affect the prediction accuracy of scPECLR (Figure S5 and STAR Methods). These results show that scPECLR can be used to infer the rate of double-stranded DNA (dsDNA) breaks at each cell division and that the rate of SCE events can vary during development.

**Figure 5:**
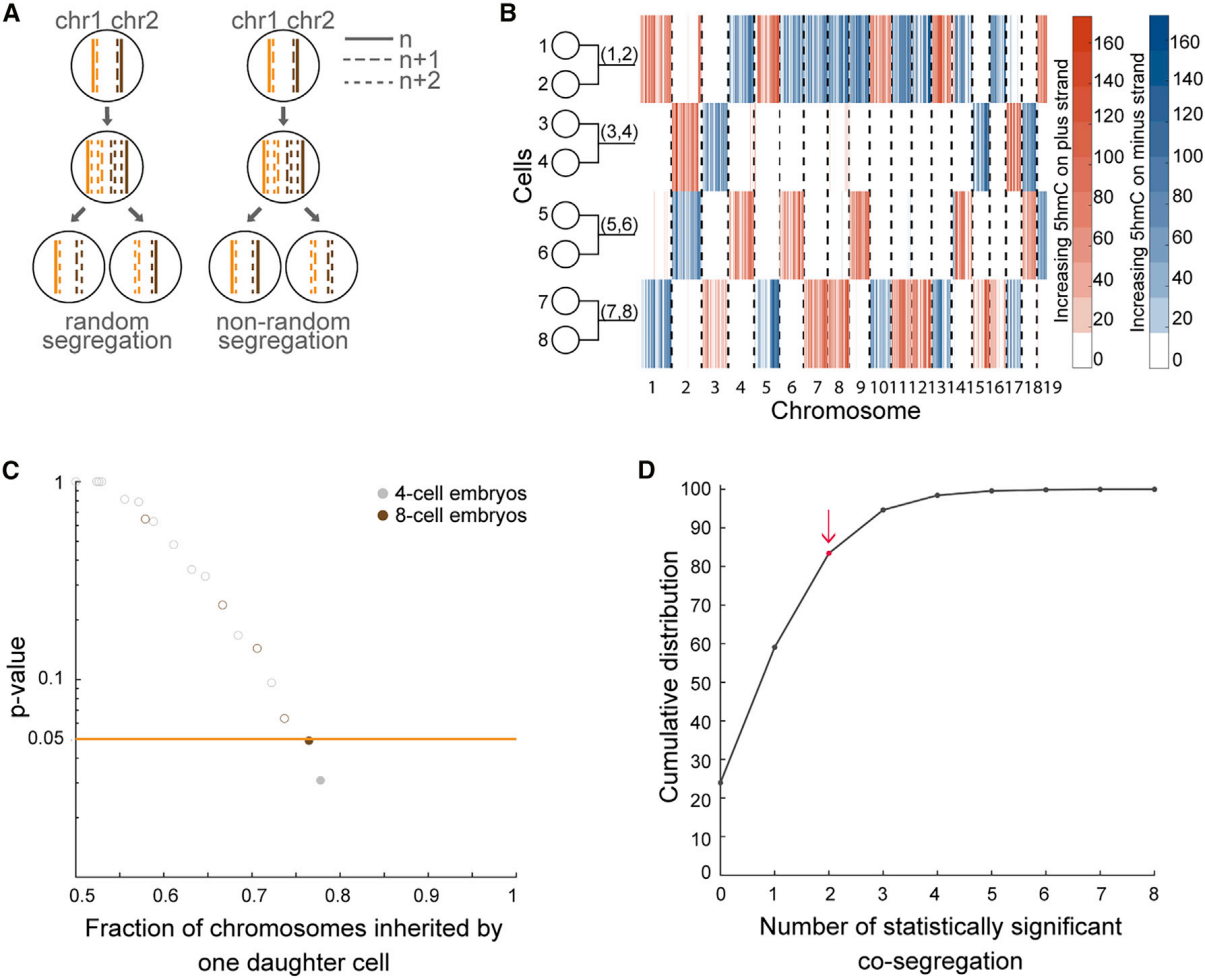
scPECLR can be used to map DNA strand segregation patterns (A) Schematic of DNA strand segregation patterns during cell division. (B) Combining the experimental 5hmC data for the 8-cell embryo in Figure 1B with the lineage tree predicted by scPECLR enables the genome-wide reconstitution of 5hmC in single cells at the 4-cell stage. (C) Testing non-random segregation of DNA strands at the 4-cell stage of mouse embryogenesis. The p values from a binomial test under a null hypothesis of random segregation shows that, out of 27 embryos, two pairs of sister cells display statistically significant (p<0.05) non-random segregation of DNA strands. (D) Twenty-seven embryos were randomly sampled 10,000 times from a pool of 100,000 simulated 4-cell embryos, generated with a constant SCE rate of b=0.3. A cumulative distribution of the number of sister pairs that display statistically significant (p<0.05) non-random segregation within the 27 embryos is shown. Red dot indicates the experimentally observed value of 2.

Finally, we explored another application of scPECLR. As scPECLR uses endogenous strand-specific 5hmC in single cells to accurately reconstruct 8-cell trees, we hypothesized that this method could quantify how paternal alleles are segregated during cell division (Figure 5A). Different stem cell populations, such as hair follicle (Huh et al., 2013), neural (Karpowicz et al., 2005), satellite muscle (Conboy et al., 2007; Rocheteau et al., 2012), and intestinal crypt stem cells (Falconer et al., 2010; Potten et al., 2002), have been shown to display non-random segregation of DNA strands that can influence cell-fate decisions. These results have led to the “immortal strand” hypothesis, which postulates that old DNA strands are retained by daughter stem cells during asymmetric cell divisions to reduce the mutational load arising from genome replication of these long-lived cells. During mouse pre-implantation development, recent reports have shown that blastomeres show biases in cell fate specification as early as the 4-cell stage (Goolam et al., 2016; White et al., 2016). Therefore, we investigated sister chromatid segregation patterns of the paternal alleles at the 4-cell stage. We first combined 5hmC data from reconstructed sister cell pairs at the 8-cell stage to generate the distribution of the oldest DNA strands at the 4-cell stage (Figure 5B). In the example shown, when comparing cells (1,2) and (3,4), the original DNA strands appear to preferentially segregate to cell (1,2). In contrast, such a non-random pattern of DNA strand segregation is not observed between sister cells (5,6) and (7,8). Quantitatively, we analyzed 14 8-cell mouse embryos (equivalent to 28 2- to 4-cell division events) to find one sister pair at the 4-cell stage that displayed statistically significant non-random segregation of DNA strands (p<0.05) (Figure 5C and STAR Methods). To directly validate these results, we performed scAba-seq on 13 4-cell mouse embryos (equivalent to 26 2- to 4-cell division events). We again observed a similar distribution with one sister pair displaying a statistically significant non-random segregation pattern of DNA strands (p<0.05), which was statistically indistinguishable from that observed in 8-cell embryos (p>0.8, two-sample KS test) (Figure 5C and STAR Methods). The observation of two non-random segregation events out of 27 embryos was not statistically significant (p>0.15), suggesting this level of non-random segregation at the 4-cell stage of mouse embryogenesis could arise by random chance (Figure 5D and STAR Methods). Thus, this study shows that strand-specific reconstruction of lineage trees can be a powerful approach to test the immortal strand hypothesis in different stem cell populations.

## Discussion

Cellular lineage reconstruction plays an important role in answering fundamental questions in several areas of biology, such as immunology, cancer biology, and developmental biology. However, most current methods have two major limitations: (1) clonal lineage reconstruction cannot establish lineage relationships at the resolution of individual cell divisions; and (2) the use of transgenes involves time-intensive generation of complex animal models and is an approach that cannot be extended to map lineages in human tissues. To overcome these limitations, we developed a generalized probabilistic framework, scPECLR, to reconstruct short-term cellular lineage trees at an individual-cell-division resolution by using strand-specific single-cell 5hmC sequencing data. Using simulated 8-cell trees, scPECLR showed a prediction accuracy of 96%. Because simultaneous live-cell imaging combined with single-cell 5hmC sequencing to directly compare lineage predictions is challenging, we validated our results by showing that 8-cell trees predicted by scPECLR, and not randomly selected incorrect trees, allow us to estimate the distribution of SCE events at the 4-cell stage that is consistent with experimental data (Figure 3A). These results highlight that scPECLR is not only accurate at reconstructing short-term lineage trees at an individual-cell-division resolution but can also be used to quantify DNA strand segregation patterns and test the immortal strand hypothesis in stem cell biology.

Furthermore, scPECLR can be applied to single-cell measurements of other non-maintained epigenetic marks, such as non-CpG methylation, 5-formylcytosine, and 5-carboxylcytosine, to reconstruct lineages (Sen et al., 2021; Wu et al., 2017), and more generally to systems where the chromosome strands present in the original cell can be distinguished from subsequently synthesized strands, such as those exposed to bromodeoxyuridine (Claussin et al., 2017; Sanders et al., 2020). Finally, we show that by integrating 5hmC data with information on genomic variants from the same cell (scH&G-seq) significantly improves the prediction accuracy of larger lineage trees. Importantly, the use of an endogenous epigenetic mark and genomic variants to reconstruct lineage trees suggests that this method can be directly extended to study human development.

### Limitations of the study

Although scPECLR enables endogenous lineage reconstruction at a single-cell-division resolution, the method suffers from two limitations. First, it cannot be applied to cell types in which the levels of 5hmC are below the detection limit of scAba-seq and scH&G-seq. However, as scPECLR relies on the relative levels of 5hmC between the two strands of a chromosome, it can be applied to many cell types, including those with low levels of 5hmC in their genome. For example, 16-cell mouse embryos display distinct mosaic genome-wide strand-specific 5hmC patterns that enable lineage reconstruction despite undergoing global erasure of DNA methylation (Messerschmidt et al., 2014; Saitou et al., 2012) (Figure 3G). A second general limitation of reconstructing larger lineage trees at an individual-cell division resolution is that the number of tree topologies increases exponentially, resulting in a drop in prediction accuracy with each additional cell division. However, as this work demonstrates, scPECLR, in combination with scH&G-seq, significantly improves the lineage-reconstruction accuracy of larger trees (Figure 4B). Finally, as most other lineage-reconstruction methods employing CRISPR/Cas9, viruses, transposons, or Cre-loxP resolve larger-scale clonal information, scPECLR presents a complementary approach to these methods for applications that require reconstructing smaller lineage trees at an individual-cell-division resolution.

## STAR★Methods

### Key resources table

**Table:** 

REAGENT or RESOURCE	SOURCE	IDENTIFIER
**Chemicals**, **peptides**, **and recombinant proteins**		

TrypLE Select Enzyme (10X), no phenol red	ThermoFisher	A12177-01
Vapor-Lock	Qiagen	981611
NEBuffer 4	New England BioLabs	B7004S
Qiagen Protease	Qiagen	19155
T4 Phage β-glucosyltransferase	New England BioLabs	M0357L
AbaSI	New England BioLabs	R0665S
T4 DNA Ligase	New England BioLabs	M0202M
Adenosine 5’-Triphosphate	New England BioLabs	P0756L
Agencourt AMPure XP	Beckman Coulter	A63880
SuperScript II Reverse Transcriptase	ThermoFisher	18064014
RNaseOUT Recombinant Ribonuclease Inhibitor	ThermoFisher	10777019
NEBNext High-Fidelity 2X PCR Master Mix	New England BioLabs	M0541L
IGEPAL CA-630	Sigma-Aldrich	I8896-50ML
AluI	New England BioLabs	R0137S
BseRI	New England BioLabs	R0581S

**Critical commercial assays**		

MEGAscript T7 Transcription Kit	ThermoFisher	AMB13345

**Deposited data**		

Single-cell 5hmC sequencing data and scH&G-seq data	This paper	GEO: GSE131678

**Experimental models**: **cell lines**		

Human: H9	WiCell	WA09

**Experimental models**: **organisms/strains**		

Mouse: C57BL/6J	The Jackson Laboratory	000664
Mouse: CBA/J	The Jackson Laboratory	000656

**Oligonucleotides**		

Double-stranded adapters for scAba-Seq	Mooijman *et al*., 2016	N/A
RandomhexRT primer	Hashimshony *et al*., 2016	N/A
Illumina sequencing primers	Hashimshony *et al*., 2016	N/A
Blunt-end adapter	scDam&T	NA

**Software and algorithms**		

scPECLR (MATLAB)	This paper	N/A
scH&G-seq	This paper	https://github.com/alexchialastri/scH-G-seq

### Resource availability

#### Lead contact

Additional information and requests for resources and reagents should be directed to and will be fulfilled by the Lead Contact, Siddharth S. Dey (sdey@ucsb.edu)

#### Materials availability

This study did not generate new unique materials nor reagents

#### Data and code availability

• The raw and processed single-cell sequencing data have been deposited at GEO and are publicly available as of the date of publication. The accession number is listed in the key resources table.
• All original code for scPECLR implementation is available in this paper’s supplemental information. All original code for scH&G-seq implementation has been deposited at GitHub and is publicly available as of the date of publication. The GitHub link is listed in the key resources table.
• Any additional information required to reanalyze the data reported in this paper is available from the lead contact upon request.

### Experimental model and subject details

#### Embryo isolation and cell picking

Embryos were gently flushed out of the infundibulum of E2.5 pregnant mice using warm M2 medium. Embryos were then manipulated in 4-ring IVF dishes coated with RNase-free BSA. Embryos were washed in PBS-0 and in Tyrode’s acid to remove the zona pellucida, then placed in a 1/3 dilution of TrypLE Select Gibco A12177-01 (stock solution is referred by Gibco as 10x concentrated) and placed on the warm plate for 2 minutes. Glass capillaries of different diameters were then used to dissociate the embryo into 2-3 clusters. Cells were then progressively extracted from each cluster, one after the other, using glass capillaries. Every single cell that is released from the clusters is immediately placed into a well of a 384-well plate containing lysis buffer.

Embryos were obtained by mating CBA and C57BL/6 mice with age ranging from 8 to 25 weeks. Mice were placed together in the evening and considered to mate at midnight (E0). The next morning, plugged females were separated. All experiments were approved by the Dutch ethical committee under the DEC KNAW HI14.2402. Mice were bred under the oversight of the animal facility of the Hubrecht Institute.

#### Cell culture and cell sorting

H9 cells were grown on Matrigel (Fisher cat #08-774-552) in mTeSR1 (Stem Cell Technologies cat #85850). Cells were passed in clumps using Versene solution (Thermo fisher scientific cat # 15040066). For sorting, cells were dissociated into single cells using TrypLE, resuspended in 1x PBS, and passed through a cell strainer.

### Method details

#### Single-cell 5hmC sequencing (scAba-Seq)

Single cells isolated from 4-, 8- and 16-cell mouse embryos were deposited into 384-well plates and the scAba-Seq protocol was performed using the Nanodrop II liquid-handing robot. Briefly, after protease treatment to strip off chromatin, 5hmC sites in the genome were glucosylated using T4-Phage β-glucosyltransferase. Next, AbaSI, which recognizes glucosylated sites and introduces double-stranded breaks with 3’ overhangs 11-13 nucleotides downstream of the recognition site, was added to the reaction mixture. The fragmented genomic DNA molecules were ligated to double-stranded adapters containing a cell barcode, 5’ Illumina adapter, and T7 promoter. The ligated molecules were amplified by *in vitro* transcription and then used to prepare Illumina libraries. A detailed protocol can be found in Mooijman et al. (2016).

#### Modeling SCE events as a Poisson process

The 5hmC data was discretized into 2 or 4 Mb bins and all SCE transitions in the 8-cell mouse embryos were identified manually. A specific SCE transition on chromosome 14 was found at the same genomic position in all embryos due to a misorientation of the reference genome (mm10), consistent with previous reports (Falconer et al., 2012; Wu et al., 2017). The stochastic nature of SCE events is modeled as a Poisson process. In using a Poisson process to model SCE events, we assume that all SCE events occur independently and at a constant rate. The probability of observing x SCE transitions in one cell cycle is given by:

(Equation 1) $$P\left[x\right]=\frac{b^{x}\text{∗}e^{-b}}{x!}$$

where b is the average number of SCE transitions per chromosome per cell division. Further, to build a probabilistic framework to reconstruct cellular lineages, we define the following parameters: (1) r is the probability that an original strand is inherited by a particular daughter cell, which is equal to ½ for randomly segregating DNA strands; (2) $k_{ij}$ is the genomic length fraction of the j^th^ segment (1≤j≤l+1, where l is the number of SCE transitions) of the original DNA strand that is observed in cell i; and (3) N is the number of unique positions where SCE events can occur.

#### scPECLR

The first step is to use the numbers of observed SCE events to estimate b using maximum likelihood estimation (MLE). Thereafter, Original Strand Segregation (OSS) analysis is used to separate the cells into two groups, reducing the number of cell divisions to be reconstructed from n to n−1. Next, within each subtree, we calculate the probability of observing a SCE pattern of a chromosome given a tree topology. For example, for the most frequently occurring pattern of one SCE event shared between two cells (see example in Figure 2A), the probability of observing it in Tree A is given by the product of the probability of having no SCE events in the first cell division and the probability of having one SCE event in the second cell division

(Equation 2) $$P\left(k_{11},\;k_{22}|\tau_{A}\right)=P_{\tau_{A}}=\left(\frac{r}{e^{b}}\right)\left(\frac{br}{e^{b}N}\right)$$

Similarly, the probability of observing this pattern in Tree B is given by the product of the probability of having one SCE event in the first cell division, and no SCE events within the original DNA strands in both cells in the second cell division

(Equation 3) $$P\left(k_{11},\;k_{22}|\tau_{B}\right)=P_{\tau_{B}}=\left(\frac{br}{e^{b}N}\right)\left(\frac{r}{e^{b}}e^{\left(\frac{\left(1-k_{11}\right)\left(N+1\right)}{N}\right)b}\right)\left(\frac{r}{e^{b}}e^{\left(\frac{\left(1-k_{22}\right)\left(N+1\right)}{N}\right)b}\right)$$

which leads to

(Equation 4) $$\frac{P_{\tau_{A}}}{P_{\tau_{B}}}=\frac{2}{e^{\frac{\text{b}}{N}}}$$

Detailed analytical expressions for the probability of observing different SCE patters are provided in the Quantification and Statistical Analysis section “Analytical expressions for the probability of observing the three most common SCE patterns”.

Subsequently, we assume that the SCE patterns on each chromosome are independent and compute the overall probability of observing SCE events over the whole genome for each tree topology. Moreover, as a 4-cell subtree has only three distinct topologies, we get

(Equation 5) $$P\left(\tau_{A}|D\right)+P\left(\tau_{B}|D\right)+P\left(\tau_{C}|D\right)=1$$

where D represents the genome-wide SCE patterns in all cells of the embryo. Rearrangement gives us the probability of observing different tree topologies given the SCE patterns over the whole genome

(Equation 6) $$P\left(\tau_{A}|D\right)=\frac{1}{1+\frac{P\left(D|\tau_{B}\right)}{P\left(D|\tau_{A}\right)}+\frac{P\left(D|\tau_{C}\right)}{P\left(D|\tau_{A}\right)}}$$

Finally, the probability of observing the topology of a particular 8-cell tree is a product of the probabilities of the two corresponding 4-cell subtrees (For details on implementing scPECLR computationally, see Methods S1: Matlab scripts for scPECLR implementation, related to STAR methods).

In 8- and 16-cell predictions, after the probabilities of all tree topologies are estimated, scPECLR assigns the topology with the highest probability as the predicted tree. Then, starting with this predicted tree, b values specific to each cell division are estimated. A second iteration with cell division-specific bvalues is then performed to obtain a new predicted tree. If the new predicted tree is not the same tree as that inferred in the first iteration, another iteration is performed starting from the predicted tree in the current iteration. This iterative process is carried out till the predicted tree is the same as that obtained in the previous iteration or until 10 iterations have been performed. In all *in vivo* mouse embryos and almost all simulated embryos, the predicted tree converges by the 3^rd^ iteration. Since we know that the iterative prediction is mostly useful when the rates of SCE events generating the simulated embryos are different for each cell division (see Quantification and Statistical Analysis section “scPECLR is robust to initial estimates of the SCE rate and to varying SCE rates at each cell division”), iterative prediction was not performed in 32-cell tree predictions to conserve computational resources.

#### Single-cell hydroxymethylation & genomic DNA sequencing (scH&G-seq)

384-well plates containing 4 μL of Vapor-Lock (Qiagen) and 200 nL of lysis buffer (0.0875% IGEPAL CA-630) are prepared and single cells are FACS sorted into each reaction well. After sorting, plates are stored at -80°C until use. The cells were lysed at 65°C for 3 minutes, and reaction wells receive 500 nL of either 1.4x Buffer4 (NEB) [negative control, scAba-Seq only], BseRI mix [1.4x Buffer 4, 0.25 units BseRI (NEB)], AluI mix [1.4x Buffer 4, 0.25 units AluI (NEB)], or a combined mixture containing both BseRI and AluI (1.4x Buffer 4, 0.125 units BseRI, 0.125 units AluI). BseRI was selected because it yields the same 2 nucleotide 3’ overhang as AbaSI, while AluI was selected because we have previously used it successfully to digest gDNA (Rooijers et al., 2019). The plate is incubated for 1 hour at 37°C followed by heat inactivation at 80°C for 20 minutes. Next, 1.8 μL of protease mix (1x Buffer 4, 6 μg Qiagen protease) is added, and the plate is heated to 50°C for 16 hours, 75°C for 20 minutes, and 80°C for 5 minutes. Then 5hmC sites in the genome are glucosylated by adding 500 nL of glucosylation mix [1x Buffer 4, 1x UDP-Glucose (NEB), 1 unit T4-BGT (NEB)] and incubated at 37°C for 16 hours. Afterwards, 500 nL of protease mix (1x Buffer 4, 2 μg Qiagen protease) is added, and the plate is heated to 50°C for 3 hours, 75°C for 20 minutes, and 80°C for 5 minutes. To detect 5hmC, 500 nL of AbaSI reaction mix (1x Buffer 4, 1 unit AbaSI) is added and the plate is incubated at 25°C for 1.5 hours, and 65°C for 25 minutes. Cells receiving the AluI mix or the combined BseRI and AluI mix have 200 μL of 64 nM blunt end adapter added as described previously (Rooijers et al., 2019). All cell also receive 200 μL of 75 nM scAba-seq adapters as described in Mooijman et al. (2016). Ligation mix [1x T4 DNA Ligase reaction buffer (NEB), 4 mM ATP (NEB), 140 units T4 DNA Ligase (NEB)] is then added to bring the total volume of each reaction well to 5 μL. Subsequently, the plate is incubated at 16°C for 16 hours. Excluding the Vapor-Lock, all reagents are dispensed using the Nanodrop II liquid handling robot. After ligation, the reaction wells are pooled and the downstream steps are performed as described previously (Gell et al., 2020; Sen et al., 2021).

### Quantification and statistical analysis

#### Analytical expressions for the probability of observing the three most common SCE patterns

Case I: The most common SCE pattern that we observed in mouse embryos is one SCE transition shared between two cells (cells 1 and 2 in Figures 2A and S1). This pattern alone cannot discriminate between sister (Tree A) or cousin (Trees B and C) cell configurations as all three topologies are consistent with the SCE pattern. Therefore, we developed a model to rigorously determine the probability of observing any SCE pattern given a tree topology. For Tree A, the probability of observing one shared SCE transition is given by the product of the probability of having no SCE events in the first cell division and the probability of having one SCE event in the second cell division. Further, there is a 1N chance that the observed SCE event occurs at a specific discretized genomic position. The probability that the original DNA strand is inherited by the mother of cells 1 and 2 is r, and the probability of inheriting the observed SCE pattern between cells 1 and 2 is given by r.

(Equation 7) $$P\left(k_{11},\;k_{22}|\tau_{A}\right)=P_{\tau_{A}}=\left(\frac{r}{e^{b}}\right)\left(\frac{br}{e^{b}N}\right)$$

Similarly, for Tree B,

(Equation 8) $$P\left(k_{11},\;k_{22}|\tau_{B}\right)=P_{\tau_{B}}=\left(\frac{br}{e^{b}N}\right)\left(\frac{r}{e^{b}}+m\right)\left(\frac{r}{e^{b}}+m\right)$$

Here, m represents the probability that the SCE events during the second cell division occur within newly synthesized DNA strands that contain undetectable levels of 5hmC. To estimate m on the left branch of the lineage tree that gives rise to cells 1 and 3, we can show that

Probability of 1 undetectable SCE transition $=\frac{br}{e^{b}}\left(1-k_{11}\right)\left(\frac{N+1}{N}\right)$

Probability of 2 undetectable SCE transitions $=\frac{b^{2}r}{e^{b}2!}{\left[\left(1-k_{11}\right)\left(\frac{N+1}{N}\right)\right]}^{2}$

Probability of n undetectable SCE transitions $=\frac{b^{n}r}{e^{b}n!}{\left[\left(1-k_{11}\right)\left(\frac{N+1}{N}\right)\right]}^{n}$

Therefore, m is given by

(Equation 9) $$\text{m}=\frac{br}{e^{b}}K_{N}+\frac{b^{2}r}{e^{b}2!}{\left(K_{N}\right)}^{2}+\frac{b^{3}r}{e^{b}3!}{\left(K_{N}\right)}^{3}+\ldots =\frac{r}{e^{b}}\left(\frac{{\left(K_{N}b\right)}^{1}}{1!}+\frac{{\left(K_{N}b\right)}^{2}}{2!}+\frac{{\left(K_{N}b\right)}^{3}}{3!}+\ldots \right)=\frac{r}{e^{b}}\left(e^{K_{N}b}-1\right)$$

where

$$K_{N}=\left(1-k_{11}\right)\left(\frac{N+1}{N}\right)$$.

Thus, (Equation 8) becomes

(Equation 10) $$P\left(k_{11},\;k_{22}|\tau_{B}\right)=P_{\tau_{B}}=\left(\frac{br}{e^{b}N}\right)\left(\frac{r}{e^{b}}e^{\left(\frac{\left(1-k_{11}\right)\left(N+1\right)}{N}\right)b}\right)\left(\frac{r}{e^{b}}e^{\left(\frac{\left(1-k_{22}\right)\left(N+1\right)}{N}\right)b}\right)$$

Further, it is trivial to show that the probability of observing the SCE pattern given Tree B or C is equal, that is

(Equation 11) $$P_{\tau_{B}}=P\left(k_{11},\;k_{22}\left|\tau_{B}\right)=P\left(k_{11},\;k_{22}\right|\tau_{C}\right)=P_{\tau_{C}}$$

Therefore, the ratio of the probability of cells 1 and 2 being sisters (Tree A) versus cousins (Trees B or C) is given by

(Equation 12) $$\frac{P_{\tau_{A}}}{P_{\tau_{B}}}=\frac{P_{\tau_{A}}}{P_{\tau_{C}}}=\frac{P_{\tau_{sisters}}}{P_{\tau_{cousins}}}=\frac{2}{e^{\frac{\text{b}}{N}}}$$

Note that the probability ratio is a function of only the SCE rate and the number of bins, and is not dependent on the location of the SCE event in this case.

Case II: Another common SCE pattern is the observation of two SCE transitions that are shared between two cells (Figures 2C, S1, and S2). For the original DNA strand to be observed in only two cells, SCE transitions must occur in the same cell cycle. Thus, the probability of observing this SCE pattern in Tree A is given by

(Equation 13) $$P\left(k_{11},\;k_{22},k_{13}|\tau_{A}\right)=P_{\tau_{A}}=\left(\frac{r}{e^{b}}\right)\left(\frac{b^{2}r}{e^{b}2!}\frac{2}{N^{2}}\right)$$

The first term is the probability that no SCE event occurs in the first cell division, and the second term is the probability of having two SCE transitions during the second cell division.

Similarly, for Tree B

(Equation 14) $$P\left(k_{11},\;k_{22},k_{13}|\tau_{B}\right)=P_{\tau_{B}}=\left(\frac{b^{2}r}{e^{b}2!}\frac{2}{N^{2}}\right)\left(\frac{r}{e^{b}}+\text{q}\right)\left(\frac{r}{e^{b}}e^{\left(\frac{\left(k_{11}+k_{13}\right)\left(N+1\right)}{N}\right)b}\right)$$

where q is the probability that undetectable SCE events occur within the 5hmC-depleted genomic region between $k_{11}$ and $k_{13}$, whose length is equal to $k_{22}$. Note that the observed SCE pattern is possible for an even number of SCE events occurring within this region. To estimate q, we can show that

Probability of 2 undetectable SCE transitions $=\frac{b^{2}r}{e^{b}2!}{\left[\frac{k_{22}\left(N+1\right)+1}{N}\right]}^{2}$

Probability of 4 undetectable SCE transitions $=\frac{b^{4}r}{e^{b}4!}{\left[\frac{k_{22}\left(N+1\right)+1}{N}\right]}^{4}$

Probability of n undetectable SCE transitions $=\frac{b^{n}r}{e^{b}n!}{\left[\frac{k_{22}\left(N+1\right)+1}{N}\right]}^{n}$

Thus, q is given by

(Equation 15) $$\text{q}=\frac{b^{2}r}{e^{b}2!}{\left(K_{N}\right)}^{2}+\frac{b^{4}r}{e^{b}4!}{\left(K_{N}\right)}^{4}+\ldots =\frac{r}{e^{b}}\left(\frac{{\left(K_{N}b\right)}^{2}}{2!}+\frac{{\left(K_{N}b\right)}^{4}}{4!}+\ldots \right)=\frac{r}{e^{b}}\left(\cosh\left(bK_{N}\right)-1\right)$$

where $K_{N}=\left[\frac{k_{22}\left(N+1\right)+1}{N}\right]$

Therefore, (Equation 14) becomes

(Equation 16) $$P_{\tau_{B}}=\left(\frac{b^{2}r}{e^{b}2!}\frac{2}{N^{2}}\right)\left(\frac{r}{e^{b}}\cosh\left(\text{b}\frac{k_{22}\left(N+1\right)+1}{N}\right)\right)\left(\frac{r}{e^{b}}e^{\left(\frac{\left(k_{11}+k_{13}\right)\left(N+1\right)}{N}\right)b}\right)$$

and the ratio of the probability of cells 1 and 2 being sisters (Tree A) versus cousins (Trees B or C) is given by

(Equation 17) $$\frac{P_{\tau_{A}}}{P_{\tau_{B}}}=\frac{P_{\tau_{A}}}{P_{\tau_{C}}}=\frac{P_{\tau_{sisters}}}{P_{\tau_{cousins}}}=\frac{2e^{(1-\frac{(1-k_{22})\left(N+1\right)}{N})b}}{\text{cosh}(b\frac{k_{22}\left(N+1\right)+1}{N})}$$

In this case, the probability ratio is a function of the genomic location of the SCE events, in addition to the SCE rate and the number of bins.

Case III: The second most common and more complicated SCE pattern occurs when an original DNA strand is shared between three cells (Figure 2C). Intuitively, Tree B with cells 1 and 3 as sisters is the least likely configuration as it requires one additional SCE transition compared to the other two trees. The probability of observing this SCE pattern in Trees A and C are given by

(Equation 18) $$P\left(k_{11},\;k_{22},k_{33}|\tau_{A}\right)=P_{\tau_{A}}=\left(\frac{br}{e^{b}N}\right)\left(\frac{br}{e^{b}N}e^{b\frac{k_{33}\left(N+1\right)}{N}}\right)\left(\frac{r}{e^{b}}e^{b\frac{(k_{11}+k_{22})\left(N+1\right)}{N}}\right)$$

(Equation 19) $$P\left(k_{11},\;k_{22},k_{33}|\tau_{C}\right)=P_{\tau_{C}}=\left(\frac{br}{e^{b}N}\right)\left(\frac{r}{e^{b}}e^{b\frac{(k_{22}+k_{33})\left(N+1\right)}{N}}\right)\left(\frac{br}{e^{b}N}e^{b\frac{k_{11}\left(N+1\right)}{N}}\right)$$

In (Equation 18), the first term accounts for one SCE event between $k_{22}$ and $k_{33}$. The second term includes one SCE event between $k_{11}$and $k_{22}$ and undetectable SCE events within the right-most genomic region, whose length is equal to $k_{33}$. The third term accounts for no SCE event within $k_{33}$ and undetectable SCE events within the left region, whose length is equal to ($k_{11}+k_{22})$. Similarly, in (Equation 19), the first term accounts for one SCE event between $k_{11}$ and $k_{22}$. The second term includes no SCE events within $k_{11}$ and undetectable SCE events within the rest of the chromosome, equivalent in length to $\left(k_{22}+k_{33}\right)$. The third term includes one SCE event between $k_{22}$ and $k_{33}$ and undetectable SCE events within the left-most genomic region. Note that Trees A and C are mirror images of each other and the probability of observing this SCE pattern is equal for these two tree configurations. For Tree B,

(Equation 20) $$P\left(k_{11},\;k_{22},k_{33}|\tau_{B}\right)=P_{\tau_{B}}=\left(\frac{b^{2}r}{e^{b}N^{2}}\right)\left(s\right)\left(\frac{r}{e^{b}}e^{b\frac{(k_{11}+k_{33})\left(N+1\right)}{N}}\right)$$

The first term is for two SCE events in the first cell division. The second term accounts for an odd number of undetectable SCE transitions within the genomic region between $k_{11}$ and $k_{33}$, such that both cells 1 and 3 contain parts of the original DNA strand. The third term includes undetectable SCE events within both left and right genomic regions, whose combined length is $\left(k_{11}+k_{33}\right)$. Further, s is given by

(Equation 21) $$s=\frac{\text{b}r}{e^{b}}{\left(K_{N}\right)}^{1}+\frac{b^{3}r}{e^{b}3!}{\left(K_{N}\right)}^{3}+\ldots =\frac{r}{e^{b}}\left(\frac{{\left(K_{N}b\right)}^{1}}{1!}+\frac{{\left(K_{N}b\right)}^{3}}{3!}+\ldots \right)=\frac{r}{e^{b}}\left(\sinh\left(bK_{N}\right)\right)$$

where $K_{N}=\left(\frac{k_{22}\left(N+1\right)+1}{N}\right)$.

Therefore, (Equation 20) becomes

(Equation 22) $$P\left(k_{11},\;k_{22},k_{33}|\tau_{B}\right)=P_{\tau_{B}}=\left(\frac{b^{2}r}{e^{b}N^{2}}\right)\left(\frac{r}{e^{b}}\sinh\left(\text{b}\frac{k_{22}\left(N+1\right)+1}{N}\right)\right)\left(\frac{r}{e^{b}}e^{b\frac{(k_{11}+k_{33})\left(N+1\right)}{N}}\right)$$

and the ratio of the probability of Tree A versus B is given by

(Equation 23) $$\frac{P_{\tau_{A}}}{P_{\tau_{B}}}=\frac{P_{\tau_{C}}}{P_{\tau_{B}}}=\frac{e^{bk_{22}\frac{N+1}{N}}}{\text{sinh}\left(b\frac{k_{22}\left(N+1\right)+1}{N}\right)}$$

Consistent with our intuition, Tree B is less likely than the other two tree topologies, and depending on the values of N,b, and $k_{22}$, Tree B can be anywhere between 2 to 100 times less likely (Figure 2C).

The approach described above can be applied to any SCE pattern. The probability of observing different SCE patterns are estimated for all chromosomes. Next, we assume that each chromosome strand is independent and compute the overall probability of observing the SCE patterns over the whole genome (D) for each Tree i ($\tau_{i}$). To determine the most likely tree, we compute and compare$\;P\left(\tau_{A}|D\right)$, $P\left(\tau_{B}|D\right)$, and $P\left(\tau_{C}|D\right)$ using Bayes’ theorem

(Equation 24) $$P\left(\tau_{i}|D\right)=\frac{P\left(D|\tau_{i}\right)\text{∗}P(\tau_{i})}{P(D)}$$

where $P\left(\tau_{i}\right)$ and P(D) are the probabilities of observing Tree i and the genome-wide SCE pattern data, respectively. $P\left(\tau_{i}\right)$ reflects prior belief of the likelihood that Tree i is the correct topology. As there are 3 possible topologies for any 4-cell tree, we get

(Equation 25) $$P\left(\tau_{A}|D\right)+P\left(\tau_{B}|D\right)+P\left(\tau_{C}|D\right)=1$$

Further, the ratio of the probability of observing Tree i versus Tree j is given by

(Equation 26) $$\frac{P(\tau_{i}|D)}{P(\tau_{j}|D)}=\frac{P(D|\tau_{i})\text{∗}P\left(\tau_{i}\right)}{P(D|\tau_{j})\text{∗}P\left(\tau_{j}\right)}=\frac{P(D|\tau_{i})}{P(D|\tau_{j})}$$

where Tree i *or* j is either Tree *A*, *B*, or *C*. The prior probabilities $P\left(\tau_{i}\right)$ are assumed to be equal to one another, a common practice in Bayesian analysis (Huelsenbeck and Ronquist, 2001). After rearrangement, we get

(Equation 27) $$P\left(\tau_{A}|D\right)=\frac{1}{1+\frac{P\left(D|\tau_{B}\right)}{P\left(D|\tau_{A}\right)}+\frac{P\left(D|\tau_{C}\right)}{P\left(D|\tau_{A}\right)}}$$

Similarly, the probability of all tree topologies can be calculated. Finally, the probability of a particular 8-cell tree is given by the product of the probabilities of the two corresponding 4-cell subtrees.

#### Simulating stand-specific 5hmC distributions

To validate the analytical expressions for the probability of observing different SCE patterns in Figures 2B and 2C, we simulated 8-cell trees where the occurrence of SCE events were modeled as a Poisson process with b=0.3 and chromosome strands were assumed to segregate randomly (r=0.5). Simulations were performed on chromosome 1 (N=97 for 2 Mb bins). These simulations were then used to estimate the probability of observing Tree A versus Tree B as a function of the position of the SCE event.

To test the accuracy of scPECLR in predicting lineage trees in Figures 3B and 4A, 8-, 16- or 32-cell embryos with 19 or 38 chromosomes were simulated as described above. All bins in the original DNA strands were hydroxymethylated whereas all subsequently synthesized DNA strands contained no 5hmC, mimicking *in vivo* experimental observations. 5,000 and 2,000 simulated trees were generated for each condition shown in Figures 3B and 4A, respectively. The trees were subsequently inputted into scPECLR to estimate the percentage of trees that are accurately predicted by the algorithm. For 16-cell trees, we also estimated the prediction accuracy of 2-, 4- and 8-cell subtrees within the full tree, and for 32-cell trees, the 16-cell subtree prediction accuracy was additionally estimated. In the 4-cell embryos in Figure 3B, as OSS accurately separates the four cells into two groups of two cells each, the lineage reconstruction problem becomes deterministic, and thus the trees are predicted with 100% accuracy. Similarly, in Figure 3C, OSS was assumed to successfully separate the two 8-cell subtrees from 16-cell trees. However, in 32-cell trees (Figure 4A), OSS could not separate cells into two groups in all embryos. Such cases would not continue forward with the calculation and would be classified as incorrect for all level of subtrees. Additionally, the prediction accuracy in 8- and 16-cell trees (Figures 3A and 3B) were bootstrapped 1000 times. The bootstrap statistics are plotted along with the prediction accuracy.

In the 32-cell tree case where half of the sister pairs are known, 8 out of the 16 sister pairs were randomly selected and became known information about the cells in each simulation. In the 32-cell tree scH&G-seq cases, the genomic variants were also modeled with a Poisson process to occur at a certain rate v per chromosome per cell division. The process starts with the first cell division (n=1, from one to two cells), which has two division actions, generating cells that are the ancestor of cells 1-16 or 17-32. If within a division action, at least one variant emerges, we would assume that we know all cells derived from that particular division action are clustered with one another. For example, if the first division action at n=1 has a variant, we would assume that we know cells 1-16 are clustered together for that simulation. Next, the step proceeds to the two cells dividing atn=2, which has four division actions. Similarly, if the third division action has a variant at n=2, cells 17-24 would be assumed to cluster together. The process continues till n=4, where there are sixteen division actions generating sister pairs. Each cell division action is treated independently. The additional information received from either half the sister pairs or the genomic variants were used to help OSS separate the cells into two groups. If cells are separated into two groups, that simulation trial would continue to the 8-cell grouping step (see “Criteria to determine 32-cell topologies to be evaluated”).

#### Consensus tree analysis

This analysis was performed on 16-cell trees to identify parts of the lineage tree that can be predicted with high confidence. The two 8-cell subtrees obtained from OSS are treated independently. The first step is to use a desired relative threshold (RT) to identify all trees that have predicted probabilities within a threshold level of the highest probability tree and include such trees for downstream analysis. All included trees are subsequently weighed equally. The second step is to examine the 4-cell subtrees of each included tree. If all trees consistently predict the same 4-cell subtree, the consensus tree includes the 4-cell subtree. This is true for most datasets as scPECLR largely predicts the 4-cell subtrees accurately in 16-cell trees (Figure 3C). When disagreement arises, if the percentage of included trees that have the same 4-cell subtree exceeds a threshold (t8), ranging from 0.55 to 1.0, the consensus tree includes the 4-cell subtree, and tree topologies that conflict with this 4-cell subtree are excluded from further analysis. If the percentage is belowt8, the consensus tree does not include the exact 4-cell subtree but instead attempts to identify as many pairs of cells as possible that appear in different 4-cell subtrees of all included trees, and the consensus analysis terminates. After the 4-cell subtrees are determined, the topology predicted within each of these subtrees is then considered. Again, if all of the remaining trees predict the same topology or if the percentage of remaining trees that predict a consistent topology exceeds a threshold (t4), ranging from 0.55 to 1.0, the consensus tree also includes that topology. Otherwise, it does not predict a specific topology within the 4-cell subtree but attempts to identify one cousin pair that appears in the 4-cell topology.

The consensus tree has different levels of specificity, ranging from predicting a full 16-cell tree, where the relationships between all cells are exact, to predicting only two 8-cell subtrees. In general, each consensus tree is constrained to contain a certain number of tree topologies, which provides information about how specific each consensus tree is. For example, in Figure 3D, the consensus tree contains six possible topologies, as there are two topologies arising from uncertainty in the subtree containing cells 5-8 and three topologies arising from uncertainty in the subtree containing cells 13-16. The lower the number of topologies contained within the consensus tree, the more specific and informative it is.

There are three parameters in the consensus tree analysis: RT, t8, and t4. RT has the largest influence on the structure of the consensus tree, while varying t8 and t4 leaves the consensus tree largely unchanged (Figures 3E, 3F, and S3) (Note: In Figure 3E, t8 and t4 are kept constant at 0.75 and 1, respectively). When the RT increases, the consensus tree becomes more specific but suffers from a higher false discovery rate (FDR). In contrast, although the effects are small, increasing t8 and t4 leads to a very modest decrease in the specificity of the consensus tree and reduction in FDR. Thus, using different parameter values allows us to tune the competing goals of specificity and accuracy of the consensus tree. In fact, for a specific FDR, there is an optimal set of parameters that gives the most specific consensus tree for a dataset. We performed a consensus tree analysis on the dataset in Figure 3B (solid blue lines), with different combinations of RT ranging from 0.05 to 0.50, and t8 and t4 ranging from 0.55 to 1.0. Each parameter set provides a consensus tree with a different level of specificity, measured by the median number of trees contained in the consensus tree, and the FDR. For any level of FDR tolerated, there is at least one parameter combination that yields the lowest median number of trees. For example, when b=0.3 and the FDR is chosen to be 30%, the optimal parameter set has RT, t8, and t4 as 0.05, 0.75, and 1, respectively, yielding the median number of trees contained within the consensus tree to be 36. Thus, for any dataset, the rate of SCE events can be estimated using MLE, and with a user-selected FDR, an optimal parameter set can be estimated to give the most specific consensus tree.

Consensus tree analysis improves the accuracy of lineage prediction in all scenarios. When the SCE rate is low (b=0.1) and the iterative prediction alone performs poorly for 16-cell trees, an error rate of greater than 99% in the iterative prediction decreases to a FDR between 30-75%. When the iterative prediction alone performs moderately (b=0.5), an error rate of ∼60% improves to a FDR between 10-45% (Figures 3B and 3E). Lastly, when the iterative prediction alone performs well (b=1.0), an error rate of ∼25% decreases to a FDR between 5-20% (Figures 3B and 3E). When b=1.0, there are only 1 to 2 median topologies contained in each consensus tree, indicating that the consensus analysis increases the accuracy of the prediction without compromising its specificity. This result shows that scPECLR and the consensus tree analysis provides a significant amount of lineage information with reasonable accuracy for 16-cell trees (Figures 3E and 3F).

To generate the consensus tree for the 16-cell embryo in Figure 3G, 1000 16-cell embryos were simulated with the same SCE rates estimated from the *in vivo* 16-cell embryo. Next, different parameter combinations of RT, t8 andt4 were used to generate consensus trees. The consensus trees were evaluated against the true tree to calculate FDR rate for each parameter combination. The lowest possible FDR rate of 15% was selected. Subsequently, the parameter combination (RT = 0.05, t8 = 0.85, t4 = 0.8) that yields the most specific consensus tree with a FDR rate under 15% was chosen for the consensus tree for the *in vivo* 16-cell embryo. There are 180 topologies contained within the consensus tree: 90 from the left 8-cell subtree and 2 from the right 8-cell subtree.

#### Criteria to determine 32-cell topologies to be evaluated

When the cells are successfully separated into two groups of 16 cells, the number of topologies to be considered reduces from more than 10^26^ to ∼4∗10^17^. We then perform “8-cell grouping”, which attempts to further split each 16-cell group into two groups of 8 cells, reducing the number of possible topologies further to fewer than 10^10^. The first step of 8-cell grouping is to consider all the possible combinations of 16 choose 8 (6435 groupings in total as cells 1-8 grouping and cells 9-16 grouping are considered one grouping). In the case where additional information about the embryos are known, the groupings that conflict with the clonal information were discarded. Next, in each grouping, the 5hmC in all cells within the two 8-cell sets were combined to generate hypothetical 5hmC data of the two cells at the 2-cell stage for that grouping. Then, the number of SCE events present in the hypothetical two cells were calculated. Only the groupings that generate the hypothetical two cells with the fewest number of SCE events were kept. The rationale is that cells accumulate SCE events on their original chromosome strands as they undergo cell division. Therefore, the fewer the SCE events present at the 2-cell stage, the more likely the 8-cell grouping is correct. The left side (cells 1-16) and right side (cells 17-32) undergo the process independently. If there are more than 30 groupings remaining in total, the process is stopped and we would conclude that tree is incorrect for 2-, 4-, and 8-cell subtree levels. The number of remaining groupings, 30, was chosen as there would be about 10000 topologies left after a successful 8-cell grouping. Then scPECLR is used calculate the probabilities of all possible topologies within the four 8-cell sets independently. The topologies that conflict with known information about the embryo are removed. Then the 8-cell sets for each grouping are combined to generate the full 32-cell tree. The grouping combination that predicts lineage of any cell more than once (i.e. one or more cell is missing from the full tree) is discarded. The probability of the full 32-cell tree is the product of the four probabilities from the 8-cell sets. The full 32-cell tree with the highest probability is the predicted tree.

#### scH&G-seq analysis pipeline

Reads were separated by their molecule type barcodes and mapped to hg19 using Burrows-Wheeler Aligner (BWA). AluI based reads were identified as described in Rooijers et al. (2019). 5hmC based reads were identified as described in Mooijman et al. (2016), with the following modification. A custom Perl script was written to identify if a read also contained a BseRI recognition site. If a read contained recognition sites for both BseRI and AbaSI, it was discarded.

#### SNP calling and processing

Variant calling was done via bcftools in a custom shell script. Briefly, the sam file was sorted and only relevant chromosomes (genomic and mitochondria chromosomes) were retained. Next, bcftools called the SNPs with default parameters. SNP calls with quality of at least 20 were kept. A custom Perl script was then used to count the occurrences of SNPs and non-SNPs for each cell at each SNP location in the sam file.

#### CNV calling and clustering

For each library, cells with fewer than 10000 reads were filtered out. 34, 50, and 31 cells passed the cut-off in the AluI, BseRI, and dual enzyme libraries, respectively. Data from autosomes were discretized into 5 Mb bins. Each bin was subsequently normalized by the number of enzyme recognition sites present within that bin. The normalized raw reads were scaled such that the median of the total data is 100. In the dual enzyme library, the normalized raw reads of AluI and BseRI were combined before scaling. The circular binary segmentation (CBS) algorithm was used to call different read count sections in each chromosome. The read count in each bin was then replaced by its mean value from the CBS algorithm. Copy number for each bin was determined by normalizing the mean read count of each cell to two copies and rounding to the nearest integer. To remove outlier bins, the bins that showed more than four copies in any cell were retroactively removed from the normalized raw read data, which was again inputted into the CBS algorithm. The read count in each bin was once again replaced by its mean value from the CBS algorithm. The copy number of each bin in each cell was subsequently recalculated. The steps were performed independently in each library. All steps of the CBS algorithm have a significance level of 0.01. All cells from the three libraries were combined, with only bins that were present in all libraries retained.

The *clValid* package in R was used to decide between hierarchical, k-means, and pam clustering algorithms and the hierarchical algorithm was recommended. The agglomerative coefficient was then used to determine the appropriate method to calculate distances between cells. Among the options: average, single, complete, and ward, the ward method was recommended. Subsequently, the *NbClust* package was used to determine the number of clusters based on the ward method and Euclidean distance. Two clusters were recommended for the combined data. A dendrogram was created based on the ward method and Euclidean distance.

#### scPECLR is robust to initial estimates of the SCE rate and to varying SCE rates at each cell division

We explored the robustness of scPECLR to initial estimates of the SCE rate by simulating strand-specific 5hmC data in 8-cell trees with a constant SCE rate (b=0.3). We then used different values of SCE rates – ranging from 0.1 to 2.0 – in scPECLR to predict the lineage tree (instead of estimating the SCE rate from the observed SCE pattern using MLE). We found that the percentage of trees that were accurately predicted did not change over the range of SCE rates, suggesting that scPECLR is robust to uncertainty in SCE rate estimation and the prediction accuracy mainly depends on the SCE rates used to generate the 5hmC data (Figures S5A and S5B).

As the 8-cell mouse embryos have varying rates of SCE events across cell divisions, we explored the robustness of scPECLR when the rates are different for each cell division. Because prediction accuracy of scPECLR is dependent on the rate of SCE events, in this analysis, we fixed the combined SCE rate (B) over 3 (or 4) cell divisions, but allowed individual cell divisions to have different rates. For 8-cell trees, the model is largely robust against varying rates of SCE events across cell divisions, with higher B and larger number of chromosomes resulting in better prediction accuracy (Figure S5C). For example, when the SCE rates are low for the first and second cell division ($b_{1}$ and $b_{2}$) and high for the third cell division ($b_{3}$), similar to the experimental observation in 8-cell mouse embryos, scPECLR predicts the lineage tree with very high accuracy (Figure S5C, H3). One case where the prediction accuracy drops modestly is when the SCE rates of the first and third cell divisions ($b_{1}$ and $b_{3}$) are low and the SCE rate of the second cell division ($b_{2}$) is high (Figure S5C, H2). In this case, the data has a large number of SCE events that are shared between cousin cells. As the SCE rate at each cell division is assumed constant during the first iteration of scPECLR, the algorithm predicts that cells sharing more SCE events are more likely to be sisters. This misidentification results in a large percentage of simulations not predicting the true tree after the first iteration. However, the prediction improves significantly after a few iterations because starting from the second iteration, the model accounts for different SCE rates at each cell division. Consequently, the varying SCE rates at each cell division has minimal impact on the accuracy of 8-cell tree prediction.

For 16-cell trees, there are a few cases where the prediction accuracy is worse than when the rates are uniformly distributed; these include situations where $b_{4}$ is low (Figure S5D, H2, H3, H13, H23, and L4). In these cases, the prediction accuracy is lower because scPECLR inaccurately infers a pair of cousin or second cousin cells as sister cells due to a large number of SCE events shared between such pairs. In contrast, cases with high $b_{4}$ values result in better prediction accuracy because scPECLR correctly identifies sister cell pairs (Figure S5D, H4, H14, H24, and H34). Finally, scPECLR also performs well when $b_{2}$ and $b_{3}$ are low as it does not misidentify cousin or second cousin pairs as sister pairs. These results suggest that in addition to the combined SCE rate, how the individual SCE rates are distributed over each cell division impacts the accuracy of reconstructing 16-cell trees.

#### Statistical test to identify non-random DNA segregation

To test the segregation pattern of DNA strands at the 4-cell stage, the 5hmC profile of 8-cell mouse embryos were combined using the lineages predicted by scPECLR to obtain the distribution of 5hmC on the original DNA strands at the 4-cell stage, while the *in vivo* experimental 4-cell mouse embryo data could be used without prior processing. If a majority of an original chromosome strand is present in one cell at the 4-cell stage, that cell is considered to inherit the entire chromosome strand. This is to account for the limited number of original strands that undergo a few SCE events during cell division. A binomial two-tailed test was conducted with a null hypothesis of random segregation (*π* = 0.5) and an alternative hypothesis of non-random segregation (*π ≠* 0.5). Two pairs of sister cells from 27 embryos were considered to display statistically significant non-random DNA segregation for p-values lower than 0.05, one pair from the 4-cell embryo dataset and the other from the 8-cell embryo dataset.

To test whether the two events of non-random segregation can be explained by chance alone, we randomly sampled 27 embryos from a pool of 100000 simulated 4-cell randomly-segregating embryos, generated with a constant SCE rate of b=0.3, and counted how many events of non-random segregation with p<0.05 were found. The random sampling was conducted 10000 times. The cumulative distribution of the number of non-random segregation events found was plotted in Figure 5D. Despite a median of one event, we failed to reject the null hypothesis that two events of non-random segregation could be explained by chance alone.

#### scPECLR implementation in MATLAB

scPECLR was implemented in MATLAB to perform iterative probabilistic reconstruction of 8- and 16-cell lineage trees. The script first uses single-cell strand-specific 5hmC data to perform OSS analysis to eliminate a majority of tree topologies. Next, it calculates the SCE rate and estimates the probabilities of all tree topologies given the genome-wide SCE pattern to predict the tree with the highest probability. Using this predicted tree, the program estimates the SCE rate for each cell division and re-calculates the probabilities of all tree topologies. The program performs iterations until the predicted tree does not change or until 10 iterations are reached. The scripts implementing scPECLR in MATLAB, along with test files, are provided as Methods S1.

## Supplemental information

Document S1. Figures S1–S5, Table S1, and Data S1

Method S1. MATLAB scripts for scPECLR implementation, related to STAR Methods

Document S2. Article plus supplemental information

## Footnotes

Supplemental information can be found online at https://doi.org/10.1016/j.crmeth.2021.100060.
