# Supplementary material for: A probabilistic framework for cellular lineage reconstruction using integrated single-cell 5-hydroxymethylcytosine and genomic DNA sequencing
Source: Cell Rep Methods. 2021 Jul 30;1(4):100060. doi: 10.1016/j.crmeth.2021.100060 (PMC8478284; doi:10.1016/j.crmeth.2021.100060)
Supplement: Document S1. Figures S1–S5, Table S1, and Data S1 [file mmc1.pdf]

Cell Reports Methods, Volume *1*

Supplemental information

A probabilistic framework for cellular lineage reconstruction using integrated single-cell 5-hydroxymethylcytosine and genomic DNA sequencing

Chatarin Wangsanuwat, Alex Chialastri, Javier F. Aldeguer, Nicolas C. Rivron, and Siddharth S. Dey

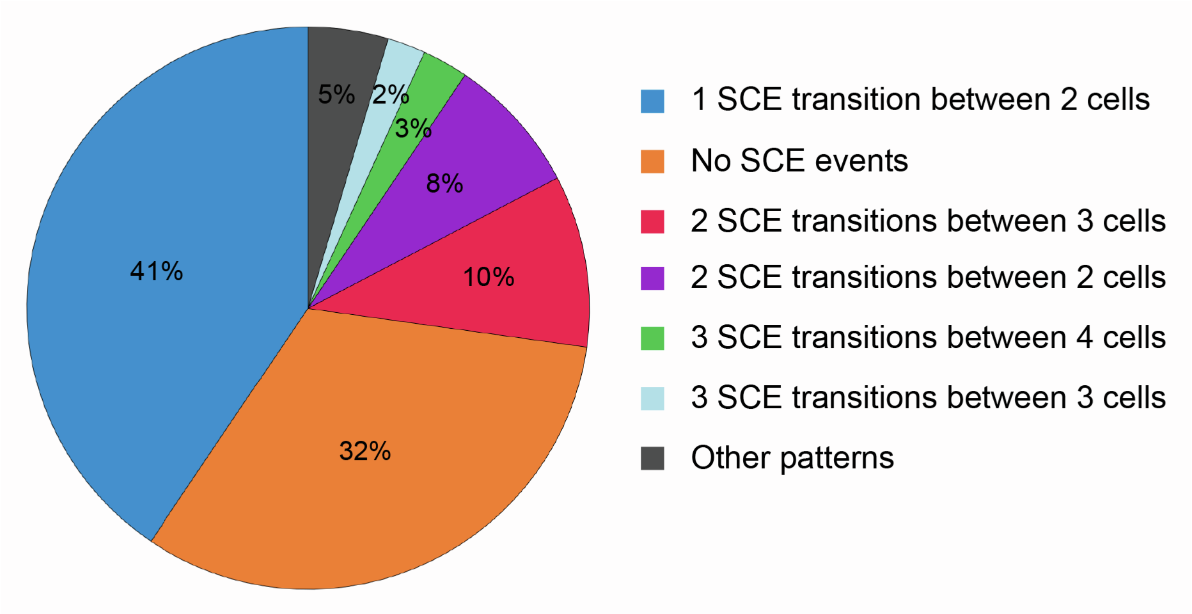

Figure S1. Distribution of SCE patterns in 8-cell mouse embryos, Related to Figure 2
Approximately 41% of the original DNA strands display one SCE transition that is shared between two cells (blue). In addition to this most frequently observed pattern, a large diversity of other SCE patterns are observed in 8-cell mouse embryos. All observed SCE patterns are used to probabilistically reconstruct cellular lineages in scPECLR. Approximately 32% of the original paternal DNA strands do not undergo SCE events in the first three cell divisions up to the 8-cell stage of mouse embryogenesis.

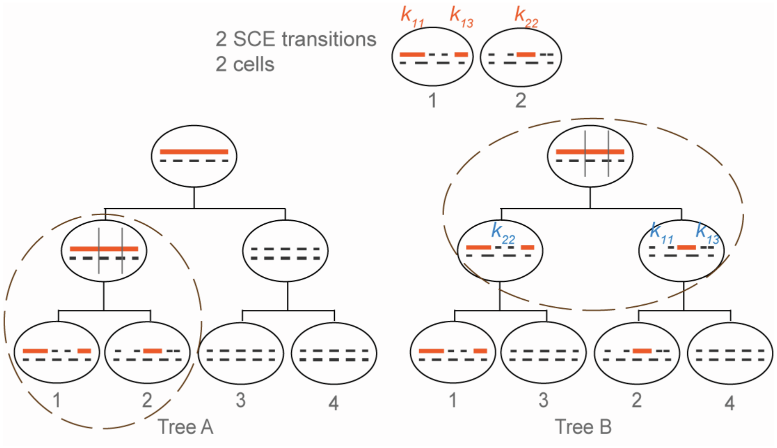

Figure S2. Reconstructing lineage trees for preimplantation mouse embryos using scPECLR, Related to Figure 2

The more complex pattern of two SCE transitions shared between two cells increasingly favors the sister tree to the cousin tree arrangement. Schematic showing the SCE events that are necessary for two cells that share two SCE transitions to be sister (Tree A) or cousin cells (Tree B). Mathematically, the original DNA strand undergoes the same number of SCE transitions in both tree topologies and the probability of observing the SCE event shown within the dotted circles is identical for Trees A and B. Further, in Tree A, the cell division that gives rise to cells 3 and 4 is unconstrained in the number of SCE events that can take place. In contrast, while any number of SCE events can occur within the k_{11} and k_{13} genomic regions in Tree B, the k_{22} region is constrained to have an even number of SCE events, thereby reducing the likelihood to observing Tree B compared to Tree A.

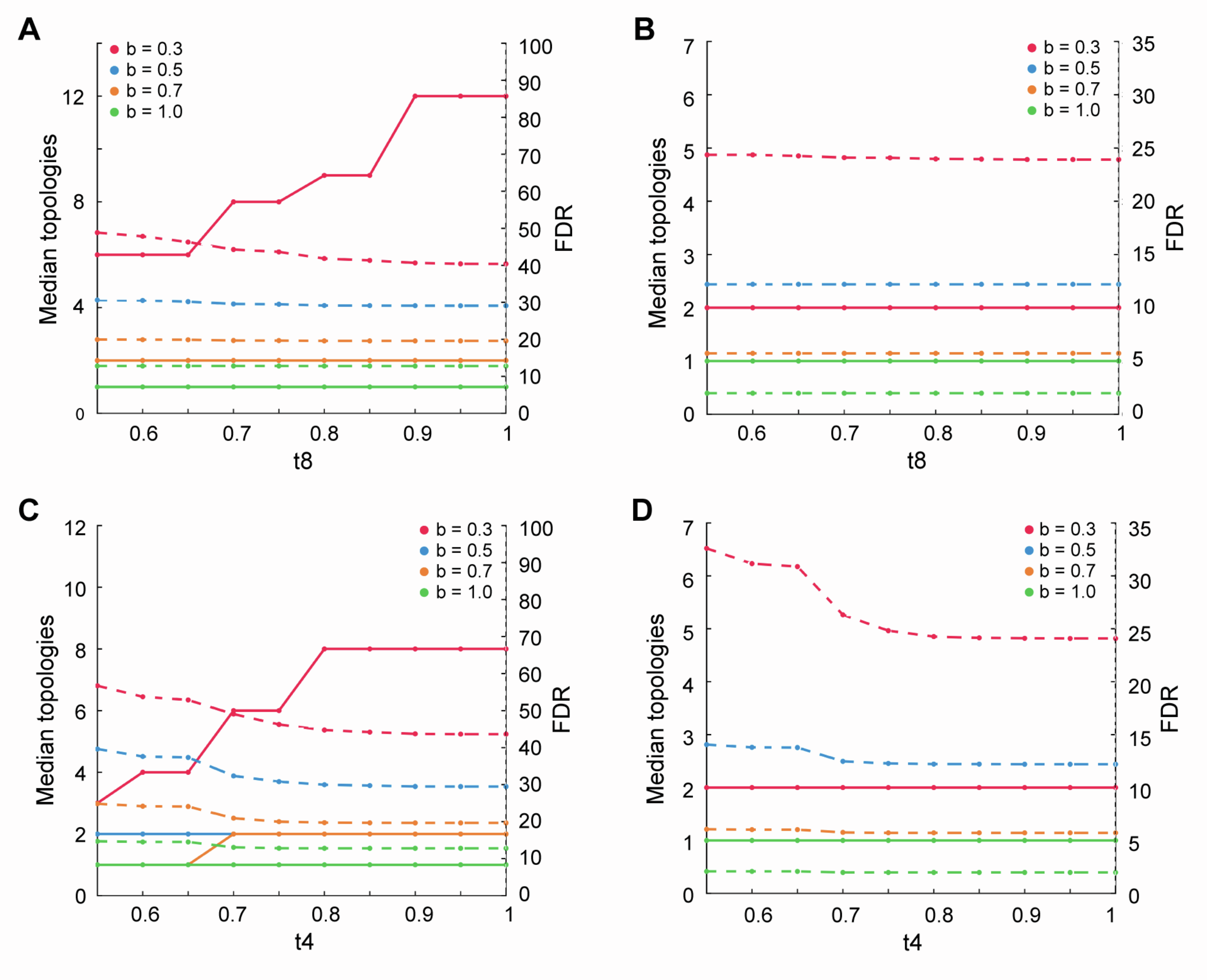

Figure S3. Parameters $t8$ and $t4$ have minor impact on the consensus tree analysis, Related to Figure 3

Panels show representative examples of how the median number of topologies of the consensus tree and the FDR varies with $t8$ and $t4$. These plots are shown for (A) $RT = 0.25$ and $t4 = 1$ for 16-cell trees with 19 chromosomes; (B) $RT = 0.25$ and $t4 = 1$ for 16-cell trees with 38 chromosomes; (C) $RT = 0.25$ and $t8 = 0.75$ for 16-cell trees with 19 chromosomes; and (D) $RT = 0.25$ and $t8 = 0.75$ for 16-cell trees with 38 chromosomes. Solid lines indicate the median number of topologies contained in the consensus tree on the left axis, and the dotted lines indicate the FDR on the right dotted axis. Varying $t8$ and $t4$ across the entire range of values shows that it does not have a significant impact on the median number of topologies contained in the consensus tree or the FDR. Note that in panel (A), the blue solid line is covered by the yellow solid line as they have the same number of median topologies in all cases. Similarly, in panels (B) and (D), both the blue and yellow solid lines are covered by the green solid line.

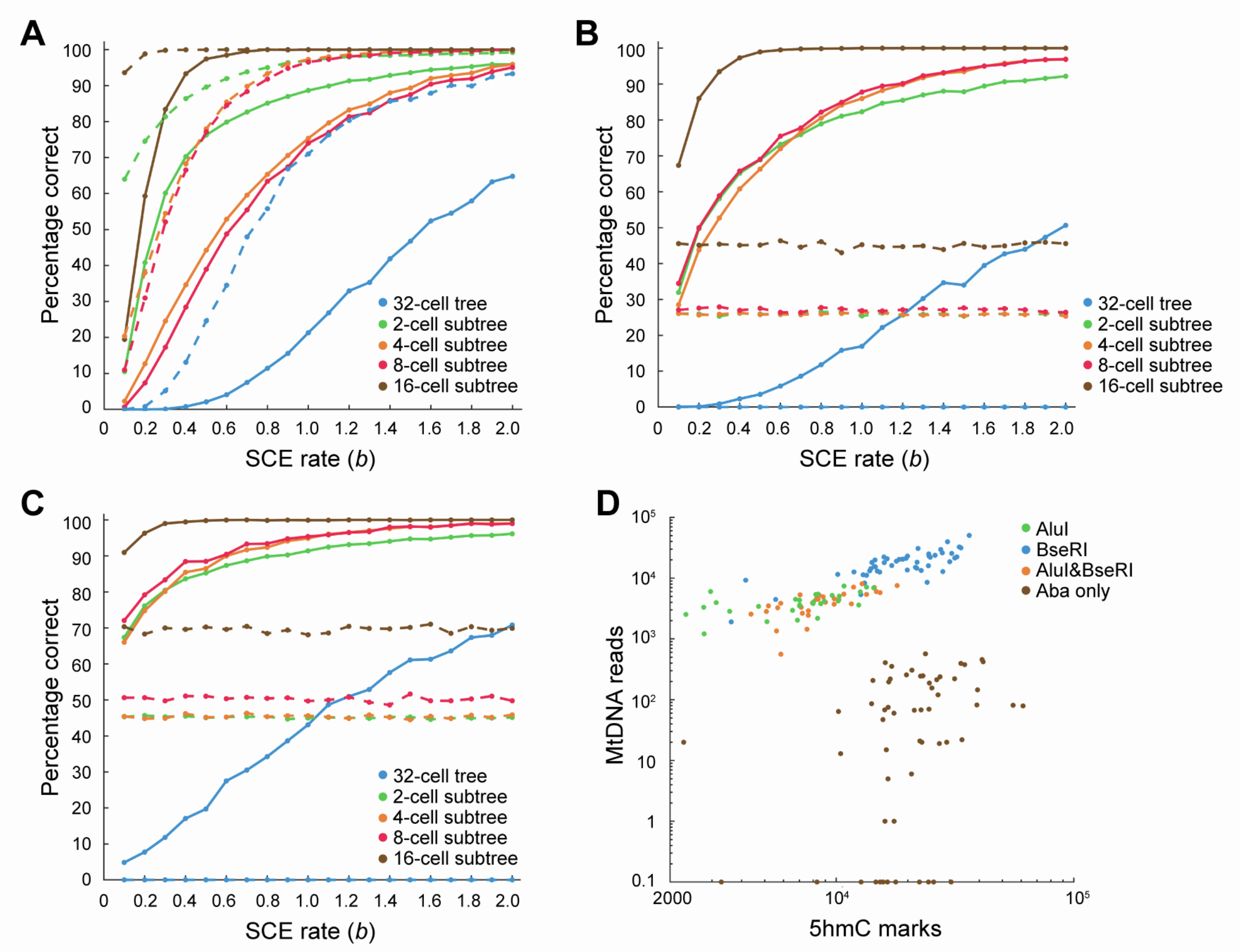

Figure S4. Combined information increases the prediction accuracy of 32-cell trees at all subtree resolutions, Related to Figure 4

(A) Panel shows the percentage of the full lineage, along with 2-, 4-, 8-, 16-cell subtrees, that are correctly predicted within simulated 32-cell trees as a function of SCE rates (*b*), assuming half of the sister pairs are known. This assumption is based on our recent work showing that strand-specific DNA methylation (5-methylcytosine or 5mC) can be used to identify half of the sister cell pairs at the 32-cell stage of mouse embryogenesis (Sen et al., 2021 Nature Communications). The prediction accuracy is computed by simulating 2000 trees. Solid and dotted lines indicate cells where 5hmC can be quantified in 19 or 38 chromosomes, respectively.

(B&C) Panel shows the percentage of the full lineage, along with its subtrees, that are accurately predicted in simulated 32-cell trees as a function of SCE rates (*b*), where the rate of genomic variants is 0.3 (B) and 0.6 (C) per chromosome per cell division. The solid lines indicate the prediction accuracy using both 5hmC and gDNA information, while the dotted lines indicate the prediction accuracy using gDNA information alone. The prediction accuracy is computed by simulating 2000 19-chr trees.

(D) scH&G-seq using AluI, BseRI, or both enzymes enable detection of mitochondria DNA reads, while maintaining similar level of 5hmC detection as scAba-seq.

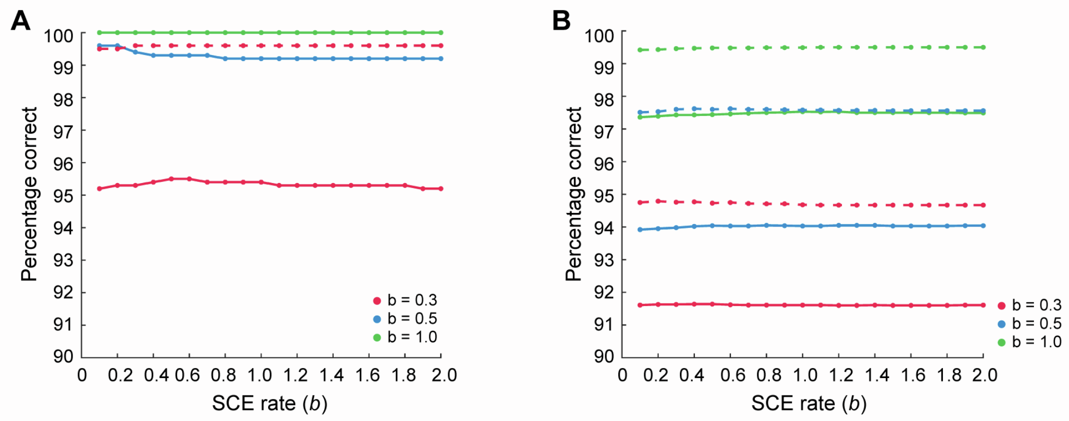

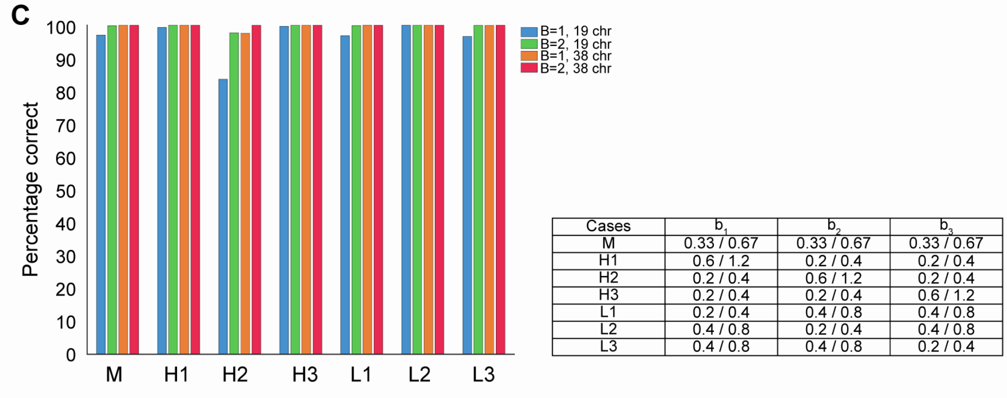

Cases	b_1	b_2	b_3
M	0.33 / 0.67	0.33 / 0.67	0.33 / 0.67
H1	0.6 / 1.2	0.2 / 0.4	0.2 / 0.4
H2	0.2 / 0.4	0.6 / 1.2	0.2 / 0.4
H3	0.2 / 0.4	0.2 / 0.4	0.6 / 1.2
L1	0.2 / 0.4	0.4 / 0.8	0.4 / 0.8
L2	0.4 / 0.8	0.2 / 0.4	0.4 / 0.8
L3	0.4 / 0.8	0.4 / 0.8	0.2 / 0.4

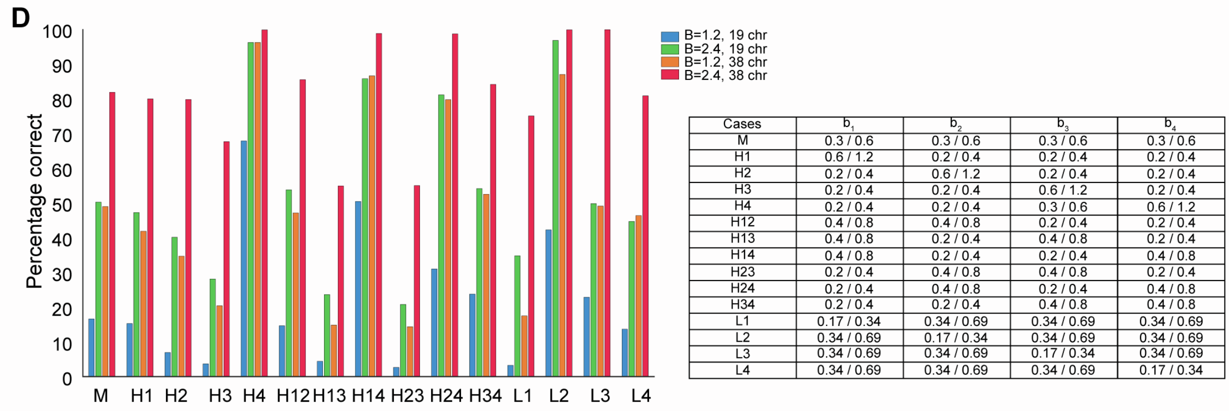

Cases	b_1	b_2	b_3	b_4
M	0.3 / 0.6	0.3 / 0.6	0.3 / 0.6	0.3 / 0.6
H1	0.6 / 1.2	0.2 / 0.4	0.2 / 0.4	0.2 / 0.4
H2	0.2 / 0.4	0.6 / 1.2	0.2 / 0.4	0.2 / 0.4
H3	0.2 / 0.4	0.2 / 0.4	0.6 / 1.2	0.2 / 0.4
H4	0.2 / 0.4	0.2 / 0.4	0.3 / 0.6	0.6 / 1.2
H12	0.4 / 0.8	0.4 / 0.8	0.2 / 0.4	0.2 / 0.4
H13	0.4 / 0.8	0.2 / 0.4	0.4 / 0.8	0.2 / 0.4
H14	0.4 / 0.8	0.2 / 0.4	0.2 / 0.4	0.4 / 0.8
H23	0.2 / 0.4	0.4 / 0.8	0.4 / 0.8	0.2 / 0.4
H24	0.2 / 0.4	0.4 / 0.8	0.2 / 0.4	0.4 / 0.8
H34	0.2 / 0.4	0.2 / 0.4	0.4 / 0.8	0.4 / 0.8
L1	0.17 / 0.34	0.34 / 0.69	0.34 / 0.69	0.34 / 0.69
L2	0.34 / 0.69	0.17 / 0.34	0.34 / 0.69	0.34 / 0.69
L3	0.34 / 0.69	0.34 / 0.69	0.17 / 0.34	0.34 / 0.69
L4	0.34 / 0.69	0.34 / 0.69	0.34 / 0.69	0.17 / 0.34

Figure S5. scPECLR is robust to initial estimates of the SCE rate and to varying SCE rates at each cell division, Related to STAR Methods

(A&B) The sensitivity of the prediction accuracy to initial estimates of the SCE rate was tested for (A) 8-cell and (B) 16-cell trees. Trees were simulated with a constant SCE rate of $b = 0.3$ (red), $b = 0.5$ (blue), $b = 1.0$ (green). To test the robustness of the algorithm, instead of estimating the SCE rate from the data, values ranging from 0.1 to 2.0 were used during the first iteration of scPECLR to predict the tree. We found that the percentage of trees that were accurately predicted was robust across the range of SCE rates for cells containing both 19 (solid lines) and 38 chromosomes (dotted lines). Note that in panel (A), the dotted lines corresponding to $b = 0.5$ and $b = 1.0$ are hidden behind the solid line corresponding to $b = 1.0$, as they all have 100% prediction accuracy for all SCE rates. These results are based on 1000 simulated trees for each condition.

(C) Panel shows the percentage of 8-cell trees (with cells containing 19 or 38 chromosomes) that are accurately predicted for varying SCE rates over the 3 cell divisions. To systematically compare the prediction accuracy of scPECLR, the combined SCE rate ($B = b_1 + b_2 + b_3$) is held constant over all cell divisions. The results show that

scPECLR accurately predicts 8-cell lineages even when the SCE rates vary with each cell division. The table denotes the SCE rates of each cell division for each condition.

(D) Panel shows the percentage of 16-cell trees (with cells containing 19 or 38 chromosomes) that are accurately predicted for varying SCE rates over the 4 cell divisions. M denotes cases where the SCE rate is constant over all cell divisions. H_i (and L_i) denotes cases where the SCE rate is higher (or lower) in the ith cell division, and H_{ij} denotes cases where the SCE rate is higher in the ith and j^{th} cell division than in the other two cell divisions. Again, the combined SCE rate is held constant. These results are based on 5000 simulated trees for each condition. The table denotes the SCE rates of each cell division for each condition.

Table S1. Mitochondrial SNPs detected in H9 cells processed using scH&G-seq, using BseRI and AbaSI enzymes, Related to Figure 4

Location	Reference Base	SNP Base	% SNP	Total Sites	Found in HT-29?*	Found in TF-1?*
73	A	G	100.0	122	Y	Y
114	C	T	100.0	120	Y	
263	A	G	100.0	178	Y	Y
497	C	T	99.4	167	Y	
686	A	G	21.3	47		
710	T	C	20.0	50		
711	T	C	20.0	50		
750	A	G	100.0	129	Y	Y
1189	T	C	99.9	12942	Y	
1438	A	G	99.7	7343	Y	Y
1811	A	G	100.0	10	Y	
2706	A	G	100.0	218	Y	Y
3105	AC	A	100.0	6		
3480	A	G	99.5	222	Y	
4769	A	G	99.9	1926	Y	Y
7028	C	T	100.0	187	Y	Y
8860	A	G	100.0	91	Y	Y
9055	G	A	100.0	1143	Y	
9698	T	C	100.0	549	Y	
10398	A	G	100.0	53	Y	Y
10550	A	G	99.6	229	Y	
10978	A	G	100.0	723	Y	
11299	T	C	100.0	1150	Y	
11467	A	G	98.7	155	Y	
11470	A	G	100.0	154	Y	
11719	G	A	100.0	4679	Y	Y
11914	G	A	100.0	138	Y	
12308	A	G	100.0	92	Y	
12372	G	A	100.0	46	Y	
12954	T	C	99.8	15205	Y	
14167	C	T	99.6	2740	Y	
14766	C	T	99.6	4966	Y	Y
14798	T	C	99.9	8627	Y	
15326	A	G	99.9	61977	Y	Y
15924	A	G	100.0	86	Y	

16224	T	C	100.0	1272	Y	
16234	C	T	100.0	1192	Y	
16311	T	C	99.8	661	Y	
16519	T	C	100.0	17	Y	

Note: “Y” indicates that these SNP sites were also reported in HT-29 and TF-1 cell lines (Diroma et al., 2020).

Data S1. Genome-wide strand-specific 5hmC distribution of 8-cell mouse embryos, Related to Figure 2

The genome-wide strand-specific 5hmC distribution of ten 8-cell and three 7-cell mouse embryos are shown. The 7-cell mouse embryos contain one blastomere from the 4-cell stage of embryogenesis that had not yet divided at the time the embryos were isolated. The mosaic pattern of 5hmC and the SCE events can be used to reconstruct the cellular lineages using scPECLR. The predicted lineage tree and the probability of observing this topology is indicated above each panel. Reconstructing the 7-cell embryos T23, T31, and T102 shows that scPECLR can be applied to non-symmetric trees. Note that for two 8-cell mouse embryos, we were not able to successfully sequence the 5hmC of one cell in each embryo (cell 1 in the embryo T11 and cell 5 in the embryo T13). By assuming that the original DNA strands that were not observed in any of the remaining 7 cells must have been present in the cell that failed to sequence, we were able to successfully predict the 8-cell lineage tree. These results suggest that scPECLR can also be used in cases where there is a limited amount of missing 5hmC sequencing data.

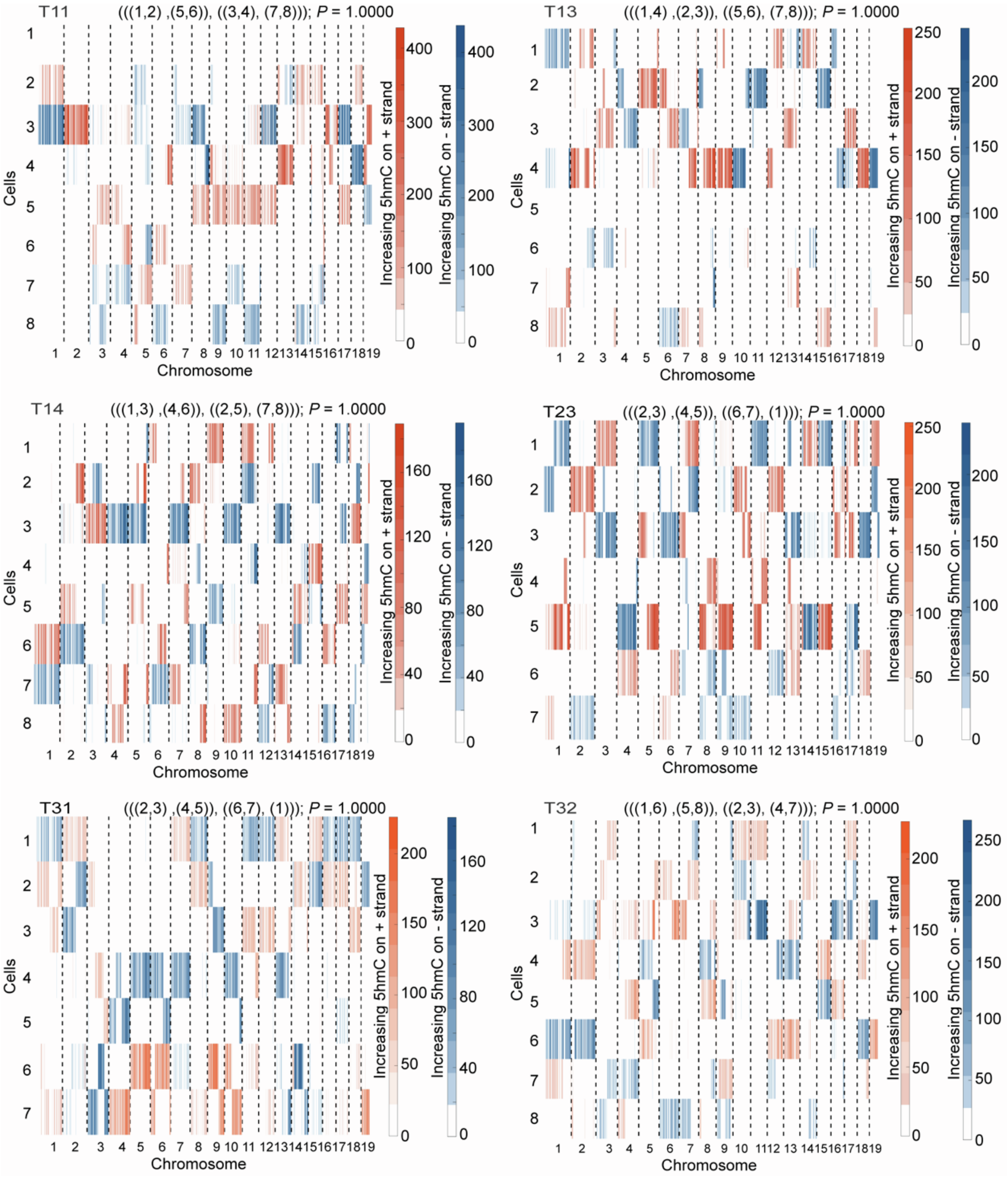

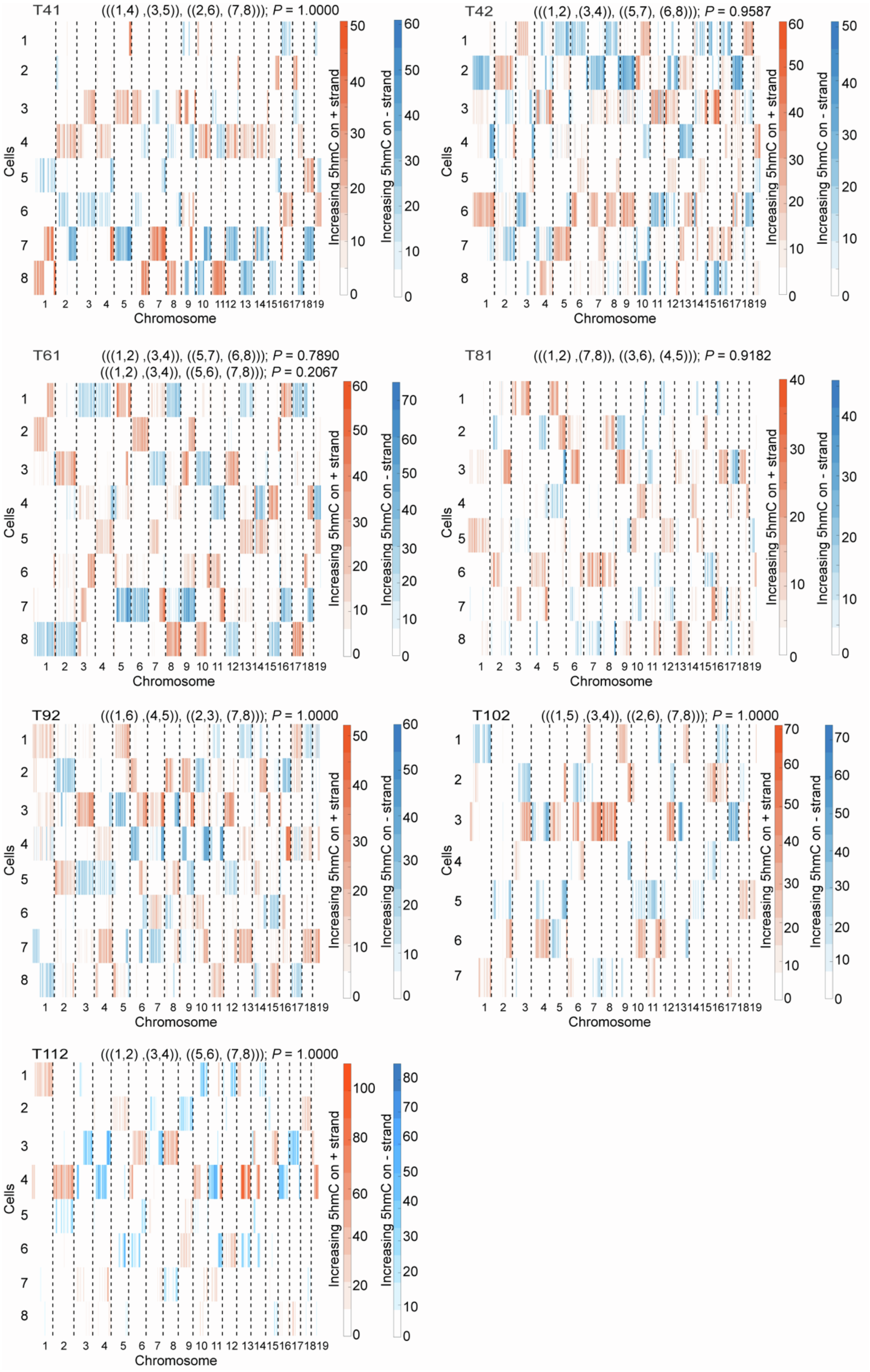
T41 (((1,4) ,(3,5)), ((2,6), (7,8))); P = 1.0000
T42 (((1,2) ,(3,4)), ((5,7), (6,8))); P = 0.9587
T61 (((1,2) ,(3,4)), ((5,7), (6,8))); P = 0.7890
(((1,2) ,(3,4)), ((5,6), (7,8))); P = 0.2067
T81 (((1,2) ,(7,8)), ((3,6), (4,5))); P = 0.9182
T92 (((1,6) ,(4,5)), ((2,3), (7,8))); P = 1.0000
T102 (((1,5) ,(3,4)), ((2,6), (7,8))); P = 1.0000
T112 (((1,2) ,(3,4)), ((5,6), (7,8))); P = 1.0000
Cells
Chromosome
Increasing 5hmC on + strand
Increasing 5hmC on - strand
